# Reconstructing metastatic seeding patterns of human cancers

**DOI:** 10.1038/ncomms14114

**Published:** 2017-01-31

**Authors:** Johannes G. Reiter, Alvin P. Makohon-Moore, Jeffrey M. Gerold, Ivana Bozic, Krishnendu Chatterjee, Christine A. Iacobuzio-Donahue, Bert Vogelstein, Martin A. Nowak

**Affiliations:** 1 Program for Evolutionary Dynamics, Harvard University, Cambridge, Massachusetts 02138, USA; 2 IST (Institute of Science and Technology) Austria, Klosterneuburg 3400, Austria; 3 The David M. Rubenstein Center for Pancreatic Cancer Research, Memorial Sloan Kettering Cancer Center, New York, New York 10065, USA; 4 Human Oncology and Pathogenesis Program, Memorial Sloan Kettering Cancer Center, New York, New York 10065, USA; 5 Department of Mathematics, Harvard University, Cambridge, Massachusetts 02138, USA; 6 Department of Pathology, Memorial Sloan Kettering Cancer Center, New York, New York 10065, USA; 7 The Sol Goldman Pancreatic Cancer Research Center, Johns Hopkins University School of Medicine, Baltimore, Maryland 21287, USA; 8 The Ludwig Center and Howard Hughes Medical Institute at The Johns Hopkins University School of Medicine, Baltimore, Maryland 21287, USA; 9 Department of Organismic and Evolutionary Biology, Harvard University, Cambridge, Massachusetts 02138, USA

## Abstract

Reconstructing the evolutionary history of metastases is critical for understanding their basic biological principles and has profound clinical implications. Genome-wide sequencing data has enabled modern phylogenomic methods to accurately dissect subclones and their phylogenies from noisy and impure bulk tumour samples at unprecedented depth. However, existing methods are not designed to infer metastatic seeding patterns. Here we develop a tool, called Treeomics, to reconstruct the phylogeny of metastases and map subclones to their anatomic locations. Treeomics infers comprehensive seeding patterns for pancreatic, ovarian, and prostate cancers. Moreover, Treeomics correctly disambiguates true seeding patterns from sequencing artifacts; 7% of variants were misclassified by conventional statistical methods. These artifacts can skew phylogenies by creating illusory tumour heterogeneity among distinct samples. *In silico* benchmarking on simulated tumour phylogenies across a wide range of sample purities (15–95%) and sequencing depths (25-800 × ) demonstrates the accuracy of Treeomics compared with existing methods.

## Abstract (web summary)

Tumours frequently metastasize to multiple anatomical sites and understanding how these different metastases evolve may be important for therapy. Here, the authors develop a method—Treeomics—that can construct phylogenies from multiple metastases from next-generation sequencing data.

Genetic evolution underlies our current understanding of cancer^1^,^2^,^3^ and the development of resistance to therapies^4^,^5^. The principles governing this evolution are still an active area of research, particularly for metastasis^6^,^7^,^8^, the final biological stage of cancer that is responsible for the vast majority of deaths from the disease. Although many insights into the nature of metastasis have emerged^9^, we do not yet know how malignant tumours evolve the potential to metastasize, nor do we know the fraction of primary tumour cells that have the potential to give rise to metastases. Moreover, the temporal, spatial and evolutionary rules governing the seeding of metastases at spatially distinct sites distant from the primary tumour have mostly remained undetermined^6^,^10^,^11^.

To better understand the evolutionary process of cancer, researchers have reconstructed the temporal evolution of patients' cancers from genome sequencing data^12^,^13^,^14^,^15^,^16^. Thus far, phylogenomic analysis has largely focused on the subclonal composition and branching patterns of primary tumours^17^,^18^,^19^. The evolutionary relationships *among* metastases are equally important but have less often been determined for several reasons^20^,^21^,^22^,^23^. First, comprehensive data sets of samples from spatially distinct metastases in different organs are rarely available. Second, most advanced cancer samples are derived from patients who have been treated with toxic and mutagenic chemotherapies, imposing a variety of unknown constraints on genetic evolution, metastatic progression and its interpretation. Third, tumours are composed of varying proportions of neoplastic and non-neoplastic cells, and inferring meaningful evolutionary patterns from such impure samples is challenging^24^,^25^. Fourth, chromosome-level changes, including losses, are frequently observed in cancers, and previously acquired variants can be lost^23^ (that is, some variants are not ‘persistent'). Fifth, even when performed at high depth, next-generation sequencing coverage is always non-uniform, resulting in different amounts of uncertainty at different loci within the same DNA sample as well as among different samples at the same locus. Finally, evolutionarily informative genetic differences among the founding cells of distant metastases tend to be rare^26^,^27^ and therefore the confidence in the inferred metastatic seeding pattern is often low.

The variety of methods that have recently been used to infer evolutionary relationships among tumours underscore these complicating factors and the need for a robust phylogenomic approach. The methods include those based on genetic distance^20^,^28^, maximum parsimony^19^,^22^,^29^, clonal ordering^3^,^15^ and variant allele frequency (VAF)^30^,^31^,^32^. Modern phylogenomic methods classify variants based on the observed VAFs, account for varying ploidy and neoplastic cell content, and reconstruct comprehensive phylogenies^33^,^34^,^35^,^36^,^37^,^38^,^39^,^40^,^41^. In this study, however, as we will show below, in the case of reconstructing the evolution of metastases, these methods suffer from the low number of informative variants and may fail to identify the subclones that gave rise to the observed seeding patterns. Classical phylogenetics assumes that the individual traits are known with certainty^24^. Consequently, these methods struggle with noisy high-throughput DNA sequencing data and do not exploit the full potential of these data due to the error-prone binary present/absent classification of variants. Furthermore, many of the methods used for inferring cancer evolutionary trees are based on those designed for more complex evolutionary processes involving sex and recombination^11^. The key conceptual difference between the new approach used here (‘Treeomics') and previous ones is that Treeomics reconstructs metastatic seeding patterns and infers the ancestral subclones that seeded metastases at various anatomic locations. Treeomics utilizes multiple samples from spatially distinct sites and assumes mostly monophyletic samples (that is, monoclonal seeding; polyclonal seeding and reseeding of metastases only happens occasionally^8^).

## Results

### Evolutionarily incompatible mutation patterns

**Figure 1:**
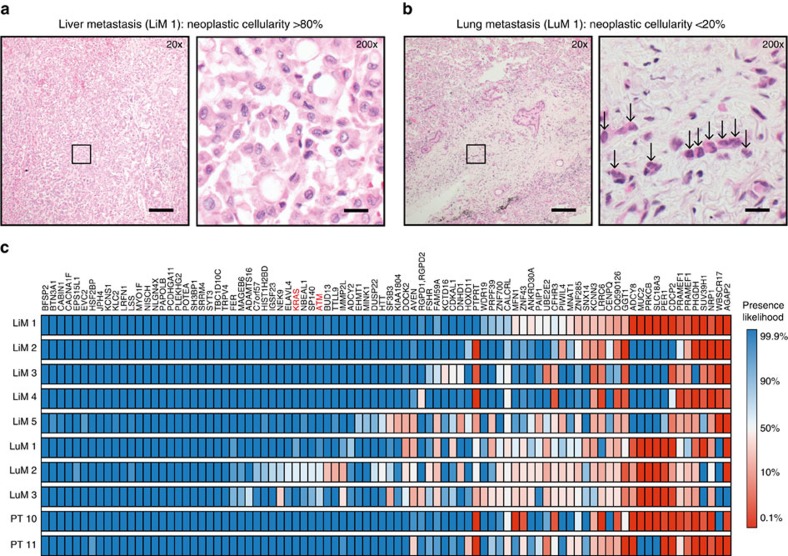
Tumour heterogeneity across lesions of pancreatic cancer patient Pam03. (**a**,**b**) Histology at low (20 × , scale bar, 200 μm) and high (200 × , scale bar, 20 μm) power of liver metastasis LiM 1 and lung metastasis LuM 1, with estimates of neoplastic cellularity determined by pathological review. Arrows highlight the few cancer cells in LuM 1. (**c**) Heatmap depicting the posterior probability (*p*) that a variant is considered as present in deep targeted sequencing data. Top five rows show samples from five distinct liver metastases (LiM 1–5); the following three rows show samples from three distinct lung metastases (LuM 1–3); the bottom two rows show different parts of the primary tumour (PT 10-11). Dark blue corresponds to a variant being present with probability >99.9% and dark red corresponds to being absent with probability >99.9%. In some samples the mutation status for the most likely clonal driver mutations in *ATM* and *KRAS* is unknown.

To illustrate our approach, we first focused on the data of a treatment-naïve pancreatic cancer patient Pam03 (ref. 27) (Fig. 1). Whole-genome sequencing (WGS; coverage: median 51 × , mean 56 × ) as well as deep targeted sequencing (coverage: median 296 × , mean 644 × ) was performed on 10 spatially distinct samples: two from the primary tumour and eight from distinct liver and lung metastases (‘Methods' section and ref. 27). Estimated purities ranged from 21 to 48% per sample (Supplementary Fig. 1), typical for low-cellularity cancers (Fig. 1). Founder variants (clonal in all samples) and unique variants (present in exactly one sample) are parsimony uninformative in the sense that they do not provide any information about common ancestors of spatially distinct samples (except the founding clone) and hence do not resolve metastatic seeding patterns. Nonetheless, unique variants can provide information about the subclonal composition and phylogeny within a sample. Parsimony-informative variants (variants present in some but not in all samples) exhibited contradicting mutation patterns when we tried to reconstruct a phylogeny consistent with the evolutionary processes underlying tumour progression using conventional methods. Identifying the evolutionarily compatible variants is known as the ‘binary maximum compatibility problem' and has been widely studied for decades^42^,^43^,^44^,^45^,^46^,^47^. A strict binary present/absent classification can be very problematic due to the above described reasons. For example, likely clonal variants in the driver genes *ATM* and *KRAS* would be classified as absent in sample LuM 2 because both were sequenced only fourteen times and were mutated only once (Fig. 1c; Supplementary Data 1). We developed a Bayesian inference model to determine the posterior probability of whether a variant was or was not found in each sequenced lesion rather than rely on a binary input (‘present' or ‘absent'; Fig. 1c; ‘Methods' section). This generalization, formalized as a Mixed Integer Linear Program (MILP)^48^, enabled us to simultaneously predict sequencing artifacts and infer phylogenies in a remarkably robust fashion.

Two clonal variants are evolutionarily compatible if there exists an evolutionary tree where each variant is only acquired once and never lost. This condition is known as the perfect (the same variant is not independently acquired twice; infinite sites model^49^) and persistent (acquired variants are not lost; no back mutation) phylogeny assumption—the basic principle of modern tumour phylogeny reconstruction methods^34^,^35^,^36^,^37^,^38^. In our case the mutation pattern of a variant is given by the set of samples where the variant is present (Supplementary Fig. 2). Therefore, two somatic variants *α* and *β* are evolutionarily incompatible if and only if samples with the following three patterns exist: (i) variant *α* is absent and *β* is present, (ii) *α* is present and *β* is absent and (iii) both variants are present. Because somatic variants are by definition absent in the germline, *α* and *β* are evolutionarily incompatible and no perfect and persistent phylogeny can explain these data (Supplementary Fig. 2). As expected, based on conventional binary present/absent classification of variants, a perfect and persistent tree consistent with the observed (noisy) data of Pam03 cannot be inferred. We show that such a phylogeny indeed exists but that it is hidden behind misleading artifacts, mostly resulting from insufficient coverage or low neoplastic cell content.

### Identifying evolutionarily compatible mutation patterns

To account for inconclusive data, we utilize a Bayesian inference model to calculate the probability that a variant is present in a sample (Fig. 1c; ‘Methods' section). Using these probabilities for each individual variant, we calculated reliability scores combining the evidence for each possible mutation pattern across all variants and samples. We constructed an evolutionary conflict graph where the nodes were determined through analysis of all mutation patterns. Each node was assigned a weight provided by the calculated reliability scores (Supplementary Fig. 3). If two nodes (mutation patterns) were evolutionarily incompatible, an edge between the corresponding nodes was added. We aimed to identify the set of nodes that maximized the sum of the weights (reliability scores) when no pair of nodes was evolutionarily incompatible. This maximal set represents the most reliable and evolutionarily compatible mutation patterns (Supplementary Methods). To evaluate the confidence in the identified evolutionarily compatible mutation patterns, we performed bootstrapping on the given variants.

### Predicting putative artifacts in sequencing data

**Figure 2:**
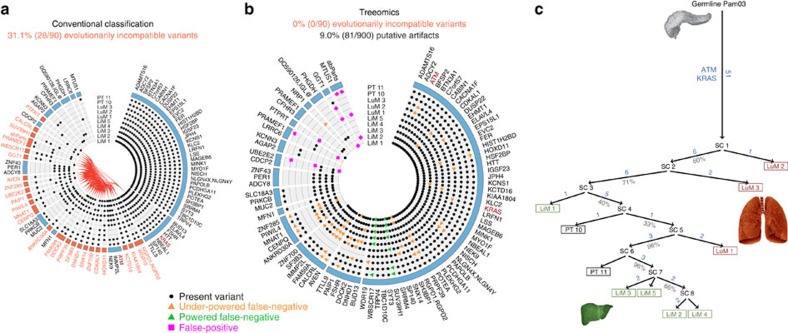
Treeomics simultaneously identified putative artifacts and inferred the evolutionary history of Pam03. (**a**,**b**) Variants shown in Fig. 1c are organized as evolutionarily defined groups (‘nodes'). Blue coloured nodes are evolutionarily compatible and red coloured nodes are evolutionarily incompatible. Based on conventional present/absent classification, 31.1% of the variants were evolutionarily incompatible (**a**). The incompatibilities are demarcated by red lines (‘edges') in the center of the circle that connect each pair of incompatible nodes. Based on a Bayesian inference model and an Integer Linear Program, Treeomics identified the most likely evolutionarily compatible mutation pattern for each variant (**b**; ‘Methods' section). This method predicted that 9% (81/900) of the variants across all samples were misclassified and thereby caused the evolutionary incompatibilities shown in panel **a**. 75% of the predicted artifacts were validated in the WGS data, among those were driver mutations in *ATM* and *KRAS*. (**c**) Reconstructed phylogeny from the identified evolutionarily compatible mutation patterns in panel **b**. Grey percentages indicate bootstrapping values from 1,000 samples. SC indicate predicted subclones. Lung metastases (LuM 1–3) are depicted in red; Liver metastases (LiM 1–5) are depicted in green; Primary tumour samples (PT 10–11) are depicted in black.

The solution obtained with the MILP directly provided the most likely evolutionarily compatible mutation pattern for each variant. By comparing our inferred classifications to conventional binary classifications, Treeomics predicted putative sequencing artifacts in the data (Fig. 2a,b). The conventional classifications differed in 9.0% of the variants in Pam03 (81 putative artifacts from 90 variants across 10 samples; Fig. 2b). As expected, the majority (68) of the differences were caused by putative false-negatives in the binary classification that were inferred to be present by Treeomics. Fifty-five of these putative false-negatives had relatively low coverage (mean: 21), explaining how they could easily be misclassified as absent given the low neoplastic cell content in the samples. Accordingly, many of these under-powered false-negatives occurred in samples with the lowest coverage (liver metastasis LiM 5, lung metastases LuM 2–3) or lowest neoplastic cell content (LuM 1; Supplementary Fig. 1). In LuM 2, the driver gene mutation *KRAS* was incorrectly classified as absent by conventional means though it is most likely a clonal founding mutation and was present at a VAF of 19% in the original WGS sample (Supplementary Table 1). Similarly, the driver gene mutation *ATM* was incorrectly classified as absent in two samples (VAF 18% and 19% in the WGS data). Although manual review of these samples revealed mutant reads in *KRAS*, it is not scalable to manually review every putative variant detected by next-generation sequencing. Some variants contained false-negatives across many samples, indicating that these variants were generally difficult to call. Remarkably, 89% (49/55) of the predicted under-powered false-negatives were either significantly present in the WGS data (38/49; mostly at higher coverage than in the targeted sequencing data), or the genomic region of the variant possessed a low alignability score^50^ (28/49; Supplementary Table 1).

For two variants sequenced at high depth, Treeomics predicted 13 putative false-negatives. The WGS data confirmed sequencing artifacts in these two variants but indicated that four likely false-positives (all absent in the WGS data) induced Treeomics to predict 13 false-negatives rather than four false-positives (Supplementary Table 2). Of the 13 putative false-positives (pink squares in Fig. 2b), 92% (12/13) were classified as absent in the original WGS data and their mean VAF was 2.3% (Supplementary Table 3). In total, 75% (49 putative false-negatives+12 putative false-positives; 61/81) of the predicted artifacts were successfully validated. Hence, we verified that at least 7% (61/900) of the variants were misclassified by conventional binary classification. If a phylogenomic method does not account for sequencing artifacts, the mutation patterns of a large fraction of variants will often be inconsistent with any inferred evolutionary tree. In Pam03, the mutation patterns of 31.1% (28/90) of the variants would be evolutionarily incompatible (Fig. 2a). These putative artifacts may also help to explain the observed high tumour heterogeneity in earlier studies and the recently reported intratumor similarity when sequencing depth is increased^19^,^26^,^27^.

### Inferring evolutionary trees

From the identified mutation patterns, Treeomics inferred an evolutionary tree rooted at the germline DNA sequence of the pancreatic cancer patient Pam03 (Fig. 2c). We found strong support for an evolutionarily related group of geographically distinct lesions: samples LiM 2–5 (liver metastases) and PT 11 (primary tumour). This result suggests that a recent parental clone of PT 11 seeded these liver metastases. We also found the same evolutionary relationship by using the low-coverage WGS data (Supplementary Fig. 4). In contrast to the targeted sequencing data, the WGS data indicated that lung metastasis LuM 1 was more closely related to LuM 2 and LuM 3. Though the low neoplastic cell content prevents a definite conclusion about the seeding subclone of LuM 1, the reconstructed phylogeny strongly suggests that the liver metastasis LiM 1 was seeded from a genetically different subclone than all other liver metastases. This diversity in seeding subclones and the origin of distinct metastases was also found in another treatment-naïve pancreatic cancer patient (Pam01) whose data similarly indicated that liver metastases were seeded from genetically distinct subclones (Supplementary Fig. 5). The phylogeny of Pam01 suggested that distinct subclones of the primary tumour gave rise to not just different liver metastases but also different lymph node metastases. This observation suggests that spatially and genetically distinct subclones in the primary tumour have the capacity to seed metastases. Moreover, these subclones are not necessarily predisposed to seeding at a particular site. In contrast, the phylogeny of Pam02 revealed that all liver metastases except one (LiM 7 with low median coverage of 27) were very closely related to each other and to various regions of the primary tumour—indicating recent divergence (Supplementary Fig. 6). Pam02's pancreatic cancer might have expanded very rapidly with only 0.5 months from diagnosis to death compared with 7 and 10 months for Pam01 and Pam03. The observed genetic similarity across geographically distinct regions of the primary tumour and seven metastases could indicate high metastatic potential of large parts of the primary tumour leading to this very short survival.

To further validate our approach, we reanalyzed data from high-grade serous ovarian cancers^20^. We were able to reproduce all phylogenetic trees of Bashashati *et al*.^20^ except for cases 1 and 5 (Supplementary Fig. 7 and Fig. 1d in ref. 20; Supplementary Fig. 8). For case 5, the authors reported an early divergence of sample 5c while Treeomics suggested a later divergence (Supplementary Fig. 7c). Comprehensive analysis of their data (reinterpreted in Supplementary Fig. 7a,b) revealed that their tree either required that several variants (including two driver gene mutations and multiple indels) occurred independently twice or that two mutations in the driver genes *ABL1* and *MDM4* were lost. Both possibilities seem unlikely (Supplementary Fig. 7 and Fig. 1d in ref. 20); this discrepancy was also identified by Popic *et al*.^37^. Treeomics did not require these implausible scenarios to construct an otherwise similar tree. Distance-based methods can be compromised by large differences in the number of acquired mutations among samples; sample 5c had twice as many mutations than all other samples. For case 1, Treeomics reported rather low bootstrap values and Popic *et al*. inferred yet another phylogeny such that no definitive conclusion could be obtained. This disagreement across methods highlights the importance of a confidence measure for the inferred branches as otherwise phylogenies are difficult to interpret in a conclusive fashion.

If multiple subclones with spatially distinct evolutionary histories (that is, polyphyletic samples due to polyclonal seeding or reseeding of a metastasis) were present in the same sample at detectable frequencies, conventional phylogenetic approaches would be unable to separate their evolutionary trajectories. In these scenarios, evolutionarily incompatible mutation patterns with high reliability scores were utilized to detect these subclones and to infer separate evolutionary histories (Supplementary Fig. 9a; ‘Methods' section). For the prostate cancer data of case 6 (ref. 17; Supplementary Fig. 9), Treeomics identified subclonal structures and separated their evolutionary trajectories without requiring high purity samples or deep sequencing data.

### *In silico* benchmarking demonstrates high accuracy

**Figure 3:**
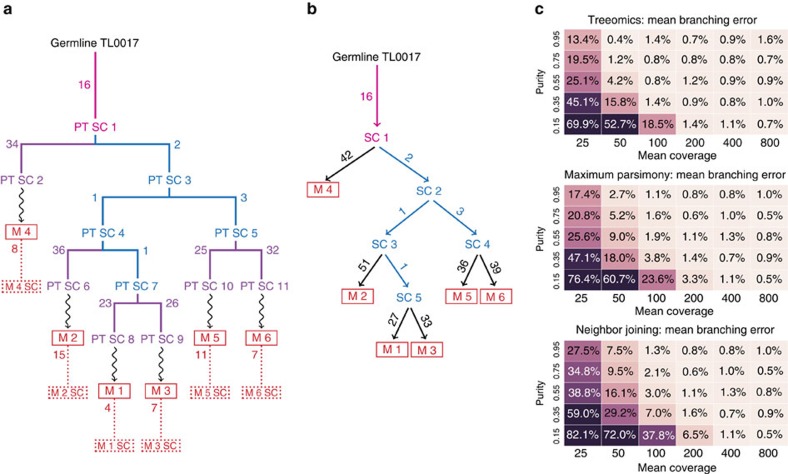
Simulated tumour phylogenies illustrate challenges in reconstructing metastatic seeding patterns. (**a**) Simulated metastatic progression according to a stochastic branching process^51^,^52^. Metastases (M 1-6) are numbered in chronological order of their seeding. Purple and blue lines indicate evolution among lineages within the primary tumour (PT). Pink numbers correspond to the founding variants present in all cancer cells and blue numbers correspond to the parsimony-informative variants. Numbers in red denote subclonal variants acquired after the seeding of the metastasis. SC indicates subclone. Dotted boxes illustrate biopsies. (**b**) Treeomics correctly reconstructed the simulated phylogeny in panel **a** by identifying the parsimony-informative variants (blue). Private mutations (purple numbers in panel **a**) acquired in the primary tumour are indistinguishable from subsequently acquired mutations (red numbers in panel **a**). (**c**) Benchmarking across 15,000 simulated phylogenies with six monophyletic metastases depicting the mean branching error conditioned on at least one variant per branch. Phylogenies reconstructed from low coverage WES data or from samples with very low neoplastic cell content exhibited high error rates independent of the used method. Necessary binary present/absent classification for maximum parsimony and neighbour joining was based on Treeomics' Bayesian inference model (variant was present if *p*>50%).

We implemented a stochastic continuous-time multi-type branching process to imitate the genetics of distinct metastases seeded according to an evolving cancer^51^,^52^ (Fig. 3; ‘Methods' section). We investigated a total of 90,000 independently simulated phylogenies comprised of 180 different combinations of sample purity, mean sequencing depth, point mutation rate, chromosome-level changes and mono- and polyphyletic metastases. Based on the simulated ground truth data, we compared the performance of Treeomics with conventional phylogenetic methods (maximum parsimony and neighbour joining) and modern phylogenomic methods (LICHeE^37^ and PhyloWGS^36^) across sample purities of 15–95% and sequencing depths of 25–800 × (Fig. 3c) representing the range of common sequencing data. A comparison of the mean branching error demonstrates that phylogenies reconstructed from low coverage whole-exome sequencing (WES) data or from samples with very low neoplastic cell content exhibit high error rates independent of the used method. For mean coverages of 100 and above, the error rates drop dramatically and phylogenies can be accurately reconstructed (Fig. 3c, Supplementary Fig. 10).

**Figure 4:**
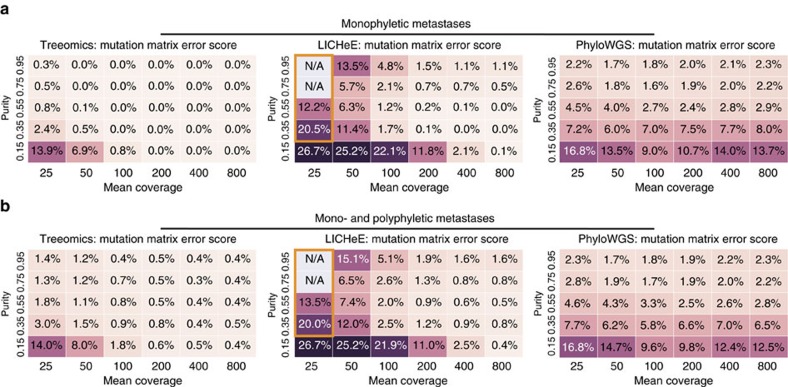
*In silico* benchmarking demonstrates the high accuracy of Treeomics across varying sample purities and mean sequencing depth. (**a**) Benchmarking across 15,000 simulated phylogenies with six monophyletic metastases (no reseeding). Treeomics greatly outperformed LICHeE in all considered scenarios. In the orange-framed scenarios, LICHeE was unable to infer a valid solution for the majority of cases. PhyloWGS exhibited mean error scores more than 10-fold higher than those of Treeomics in most considered scenarios. (**b**) Benchmarking across 15,000 simulated phylogenies with three monophyletic and three polyphyletic metastases imitating patients with reseeded metastases^21^,^23^,^53^. Treeomics exhibited the lowest mean error score across all scenarios. The performance of PhyloWGS did not significantly change compared with monophyletic metastases (possibly due to the advantageous input). The error scores of Treeomics and LICHeE slightly increased.

Current subclone inference algorithms do not directly reconstruct phylogenies of distinct sites as Treeomics does but infer joint phylogenies of variants, which are sometimes simultaneously grouped into subclones^36^,^37^,^38^,^39^,^40^. To enable a comparison of these slightly different methodologies, we developed a mutation matrix error score (similar as in ref. 37) that checks (i) if variants of the same subclone were indeed assigned to the same subclone and (ii) if the ancestral relationship among variants was correctly determined (‘Methods' section). For example, in the simulated phylogeny illustrated in Fig. 3a, the tested tools had to correctly assign the acquired variants to the founding subclone (PT SC 1) and the parsimony-informative subclones (PT SC 3-5, 7). Since the runtime of PhyloWGS increases significantly with the number of variants, we removed all private variants in the input for PhyloWGS (purple and red variants in Fig. 3a). Treeomics and LICHeE were provided with all detected variants and therefore had to distinguish between parsimony-informative variants and private variants as well as sequencing artifacts. All tools accurately identified ancestral subclones and their variants for mean coverages above 200 and a neoplastic cell content >35% (Fig. 4a). Treeomics outperformed LICHeE and PhyloWGS in all considered scenarios (Fig. 4a). In the majority of scenarios, the error score of PhyloWGS was more than 10-fold higher than the error score of Treeomics. For mean coverages below 50, the error score of LICHeE increased notably while PhyloWGS was mostly struggling with low neoplastic cell content (<35%).

In the case of reseeded metastases^21^,^23^,^53^ leading to multiple evolutionary trajectories and therefore polyphyletic lesions, the error score of Treeomics and LICHeE slightly increased while the performance of PhyloWGS did not change significantly (possibly due to the advantageous input; Fig. 4b). Treeomics exhibited the lowest error score across methods in all scenarios. Interestingly both Treeomics and LICHeE performed best in the case of high sequencing depth but low or medium purity—suggesting that there is further room for improvement (Fig. 4b). We hypothesize that the higher purity leads to more detected private variants and hence to more potential sequencing artifacts. In the case of an elevated point mutation rate (for example, due to mismatch repair deficiency) or highly chromosomally unstable cancers^54^, Treeomics continued to have the lowest mutation matrix error score in 119 of 120 considered scenarios (Supplementary Figs 11 and 12). The runtime of PhyloWGS was around 5-8 h per simulated phylogeny (in total ∼300,000 core computing hours; elevated mutation rate could not be evaluated due to the high runtime), while LICHeE needed on average a few minutes (∼4,000 h) and Treeomics less than a minute per case (in total ∼800 core computing hours).

## Discussion

The new approach described here efficiently reconstructs the evolutionary history, detects potential artifacts in noisy sequencing data, and finds the ancestral subclones giving rise to the distinct metastases. The evolutionary theory of asexually evolving populations combined with Bayesian inference and Integer Linear Programming enabled us to infer detailed phylogenomic trees with significantly fewer errors than existing methods (Figs 3 and 4, Supplementary Figs 10–12). In contrast to other tools, Treeomics accounts for putative artifacts in sequencing data and can thereby infer the branches where somatic variants were acquired as well as where some may have been lost during evolution, presumably through losses of heterozygosity resulting from chromosomal instability^23^,^55^. The branching in the inferred trees shed new light on the origin and the seeding patterns of particular metastatic lesions^6^,^11^. For example, in contrast to colon cancer, where liver metastases are assumed to seed lung metastases^56^, our results suggest that this may not be the case in pancreatic cancer. The reconstructed phylogenies also indicate that distinct subclones in the primary tumour were equally capable to seed metastases in the same and in different organs (Supplementary Fig. 5). However, we did not find any evidence for polyphyletic metastases, which confirms findings in a mouse model of pancreatic cancer where the large majority of lung and liver metastases were monophyletic^53^. The evolutionary rules of natural metastatic cancers leading to the highly non-random pattern of metastases in Pam03 are just beginning to emerge.

Despite these detailed reconstructed phylogenies, there are several limitations that should not be neglected. A low mutation matrix error score does not directly imply correctly reconstructed seeding patterns (compare Figs 3c and 4a). A method can exhibit low mutation matrix error scores while exhibiting high branching errors and vice versa. Moreover, without additional data, even correctly inferred cancer phylogenies do not directly provide information about the temporal ordering in which metastases were seeded nor about the anatomic location of the seeding subclones. For example, metastasis M4 diverged first in the simulated phylogeny but was seeded rather late (Fig. 3a). Furthermore, a single seeding event cannot be distinguished from multiple seeding events from the topology of the reconstructed tree alone^11^. Only sufficient sampling of all sites can provide evidence about the location of the seeding subclone and the likely timing of the seeding event. For example, the genetic similarity of the primary tumour sample PT 11 and the liver metastases LiM 2–5 suggests multiple seeding events from a recent ancestor of PT 11. Future phylogenomic approaches could incorporate estimated growth rates and mutation rates to better quantify the probability of metastasis-to-metastasis spread.

We have designed Treeomics from first principles to directly handle ambiguity in high-throughput sequencing data, including samples with low neoplastic cell content or coverage. The mutation patterns and their evolutionary conflict graph form a robust data structure and consequently the painful task of semi-automatic filtering becomes unnecessary. As a result of the Bayesian confidence estimates for the individual variants, this method can infer more robust results than traditional phylogenetic methods, which employ a binary representation of sequencing data (Fig. 2a). Furthermore, as shown above, distance-based methods can produce results inconsistent with the evolutionary theory of cancer as they often ignore knowledge of biological phenomena specific to neoplasia (Supplementary Fig. 7). We note that PhyloWGS, LICHeE and other subclone inference methods have not been designed to reconstruct phylogenies based on these few genetic variants that determine the evolutionary history of metastases. The key difference between these approaches is that Treeomics assumes that mixing of subclones from two spatially distinct sites and hence polyphyletic samples are rare^23^,^26^,^53^. Treeomics therefore works extremely well among metastases but is not applicable for liquid cancers. On the contrary, tools like PhyloWGS work extremely well in liquid cancers. Last, we compared our results to AncesTree^38^, which roughly identified the evolutionarily related samples in Pam03 but excluded 70% (63/90) of the variants (among them the driver gene mutations in *KRAS* and *ATM*) in the inferred phylogeny due to evolutionary incompatibilities (Supplementary Fig. 13).

At present, Treeomics only employs nucleotide substitutions and short insertions and deletions—a subset of the available information. The benchmarking results demonstrate that a single mutation varying in two samples is typically sufficient for Treeomics to infer the correct evolutionary history (Fig. 3a,b); a crucial property given the high genetic similarity of metastases^26^,^27^. Other types of data, such as copy number alterations, structural variations and DNA methylation, could be incorporated into Treeomics to further improve the accuracy of the inferred results.

## Methods

### DNA sequencing design and validation

Sequencing data were generated in two stages^27^. First, genomic DNA from 26 tumour samples of three subjects (20 metastases and six primary tumour sections) was evaluated by 60 × whole-genome sequencing (WGS) using an Illumina Hi-Seq 2000 (Fig. 1, Supplementary Figs 5 and 6 for anatomic locations of the individual samples). Importantly, genomic DNA from the normal tissue of each patient was used to facilitate identification of somatic variants. We obtained an average coverage of 69 × with 97.5% of bases covered at >10 × , revealing a total of 127,597 putative coding and noncoding somatic mutations (average of 4,908 per sample). To limit the artifacts generated by WGS and alignment, we filtered the putative variants using several quality parameters, including read directionality, mutant allele frequency detected in the normal, known human SNPs, and the number of independent tags at each site. This analysis, combined with manual inspection of the raw data, yielded a total of 2,105 potential mutations for subsequent validation.

Second, we utilized a targeted sequencing approach to independently screen every mutation that we observed to be of high quality in at least one WGS tumour sample. Briefly, probes for capture were designed to flank each potential mutant base (2,105) and libraries were prepared for the original 26 WGS samples of the three subjects. Using an Illumina chip-based approach, we successfully aligned, processed, and validated 381 mutations (range 106–164 per patient) at an average sequencing depth of 731 × (Supplementary Data 1–3). In addition to the increased coverage and sensitivity of targeted sequencing, both sequencing approaches generated independent data sets in which we could directly compare putative variants *in silico* among many tumours within a patient. Additional details regarding patient selection, processing of tissue samples and DNA extraction and quantification can be found in ref. 27.

### Bayesian inference model

To compute reliability scores for each mutation pattern, we extract posterior probabilities for the presence and absence of a variant in a sample from a Bayesian binomial likelihood model of error-prone sequencing. If *f* is the true fraction of variant reads in the sample, *π* is our prior belief about *f,* and *e* is the sequencing error rate, the posterior distribution *P* of *f* given *N* total reads and *K* variant reads is


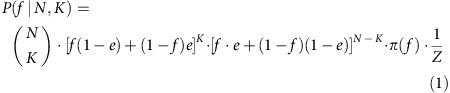


where *Z* is a normalizing constant (Supplementary Methods). A priori, the VAF in a sample is exactly zero (*f*=0) with some positive probability *c*_0_. The prior *π* is then of the following form


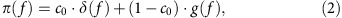


where *δ*(*f*) denotes the Dirac delta function and *g*(*f*) denotes a prior given the variant is present. We use a sample-specific prior function to account for the by multiple fold varying neoplastic cell content across samples (Supplementary Methods; Supplementary Fig. 2). The posterior probability that a variant is absent in a sample with low neoplastic cell content will be lower than in a sample with high neoplastic cell content despite the same *K* and *N* (Supplementary Methods). The posterior probability that a variant is absent, denoted by *q*, and the probability that a variant is present, denoted by *p*, are


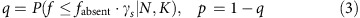


where *γ*_*s*_ is the estimated neoplastic cell content in sample *s* and *f*_absent_ is the maximal frequency threshold for an absent single nucleotide variant (SNV) (Supplementary Methods). A variety of more sophisticated variant detection algorithms can be used here as long as the output can be converted to posterior probabilities of presence and absence. We obtained robust results across all investigated scenarios with the frequency threshold of *f*_absent_=0.05. We calculate the probability of each mutation pattern for a particular variant by multiplying the corresponding posterior probabilities for each sample. Each mutation pattern has some positive probability, but those supported by the data are given much more weight. A mutation pattern *v* is denoted as a binary vector of length |*S*| (total number of samples) where *v*_*s*_ is 1 if the variant is present in sample *s* and 0 if absent. The likelihood *L*_*μ*_(*v*) that a variant *μ* exhibits pattern *v* is


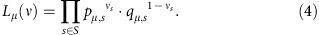


If the presence or absence of a variant in some samples is uncertain, the likelihood of any individual mutation pattern will generally be lower. The reliability score *ω*_*v*_ of each mutation pattern *v* (corresponding to a node in the evolutionary conflict graph; Supplementary Fig. 3) is given by


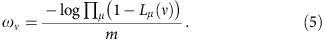


Assuming mutations are independent across each other and across samples, the argument of the logarithm denotes the likelihood that no mutation has pattern *v* and hence leverages the full sequencing information from all variants. With these scores (weights) normalized by the number of considered variants *m*, the minimum weight vertex cover of the evolutionary conflict graph corresponds to identifying the most reliable and evolutionarily compatible mutation patterns (see Supplementary Methods for further details).

### Identifying evolutionarily compatible mutation patterns

Given the calculated reliability scores, we efficiently find the most reliable and evolutionarily compatible mutation pattern for all variants via solving a MILP^48^. In the Supplementary Information we prove that finding these mutation patterns is equivalent to solving the Minimum Vertex Cover problem; one of Karp's original 21 *NP*-complete problems^42^,^57^. In the Minimum Vertex Cover problem one wants to find the minimum set of nodes in an undirected graph such that each edge in the graph is adjacent to one of the nodes in the minimum set. Therefore, by definition all edges are covered by the nodes in the minimum set. Similarly, we try to find the weighted set of nodes (here mutation patterns) with the minimal sum of reliability scores such that no evolutionary incompatibilities in the conflict graph remain. After this minimal set of nodes and their adjacent edges have been removed from the graph, we can easily infer an evolutionary tree since evolutionary conflicts no longer exist among the remaining nodes (that is, all edges were covered and removed with the minimal set). The remaining set of mutation patterns is by definition the maximal set of evolutionarily compatible patterns (Supplementary Methods).

In the evolutionary conflict graph *G*=(*V*, *E*), each node *i*∈*V* represents a different mutation pattern. For *n* samples, the number of nodes |*V*| is given by 2^*n*^. For each pair of evolutionarily incompatible mutation patterns *i* and *j*, there exists an edge (*i*, *j*)∈*E*. The weight (*c*_*i*_) of each node *i* is given by the reliability scores *ω*_*i*_ described in the Bayesian inference model section (Supplementary Fig. 3).

The MILP to find the minimal-weighted set of evolutionarily incompatible mutation patterns is defined by the following objective function and constraints:


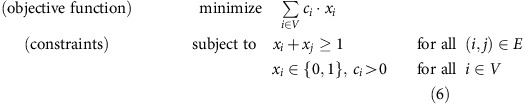


This formulation guarantees that the MILP solver finds the minimal value of the objective function such that all constraints are met and hence the nodes in the selected set cover all edges. The evolutionarily compatible and most reliable mutation patterns {*i*|*x*_*i*_=0} are given by the complement set of the optimal solution {*i*|*x*_*i*_=1} to the MILP.

Day and Sankoff showed that inferring the most likely evolutionary trajectories is a computationally challenging problem (*NP*-complete^42^). Sophisticated approximation algorithms have been developed in the context of language and cancer evolution^43^,^45^,^46^. However, medium-sized instances of *NP*-complete problems are no longer intractable due to the enormous engineering and research effort that has been devoted to ILP solvers. The MILP^48^ formulation enables an efficient and robust analysis of large data sets. We prove that an approximation algorithm that would guarantee that its solution is at most 36.06% worse than the optimal solution cannot exist unless the complexity class *P=NP* (Supplementary Methods, Theorem 1). Salari *et al*.^46^ explored a related approach but approximated two *NP*-complete problems, possibly leading to suboptimal results. Treeomics produces a mathematically guaranteed to be optimal result without convergence or termination issues. Note that a mathematical optimal solution is not necessarily equivalent to the biological truth, especially in the case of low neoplastic cell content or coverage (Figs 3 and 4). MILPs may also be useful in other areas of phylogenetic inference where methods with strong biological assumptions (for example, constant mutation rates or specific substitution profiles) are not applicable or are computationally too expensive to obtain guaranteed optimal solutions.

### Inferring evolutionary trees

After the evolutionarily compatible mutation patterns {*i*|*x*_*i*_=0} have been identified and variants are assigned to their most likely evolutionarily compatible pattern based on the maximum likelihood weights given by the Bayesian inference model, the derivation of an evolutionary tree is a trivial computational task. In quadratic time (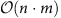) of the input size we construct a unique phylogeny where *n* is the number of samples and *m* is the total number of distinct variants^58^. The branches where the individual variants are acquired follow from the inferred tree.

### Detecting subclones of distinct origin

Evolutionary incompatible mutation patterns with high reliability scores may indicate mixed subclones with distinct evolutionary trajectories (Supplementary Fig. 9). Recall that evolutionary incompatibility requires that the conflicting variants need to be present together in at least one sample. However, even if both variants are mutated in a statistically significant fraction in the same sample, these variants may not be present in the same cells and the evolutionary laws of an asexually evolving population may not be violated. If an evolutionarily incompatible mutation pattern exhibits a reliability score higher than expected from noise, Treeomics utilizes this evidence to infer subclones with distinct evolutionary trajectories and unidirectional spreading. A detailed pseudo-code is provided in the Supplementary Methods. Subsets (descendants) and supersets (ancestors) of the conflicting mutation pattern are simultaneously identified and a comprehensive evolutionary tree is inferred. We performed extensive benchmarking of the subclone detection algorithm for various scenarios described in the following section (Fig. 4, Supplementary Fig. 9). Furthermore, we tested the method on sequencing samples from the same prostate. After two subclones were separated in mixed samples from a prostate tumour^17^, 12,643 (out of 12,645) variants supported the inferred evolutionary tree (Supplementary Fig. 9). The remaining two variants were predicted to be false-positives by Treeomics.

### *In silico* benchmarking

To assess the performance of Treeomics, we simulated metastatic progression according to a stochastic multi-type continuous-time branching process^51^,^59^,^60^,^61^,^62^,^63^ where metastases are seeded independently at random. Cells divide with birth rate *b*=0.16, die with death rate *d*=0.1555, and can leave the current site to successfully colonize a new site with probability *q*=10^−9^, (refs 51, 64). When a cell divides, a point mutation is acquired with probability *u*=0.145 (assuming a point mutation rate of 5 × 10^−10^ per basepair and 45 megabases covered by Illumina exome sequencing^65^) and a copy number variant (CNVs) is acquired with a rate of 0.1% per division. The evolutionary process is initiated by a single advanced cancer that already accumulated driver gene mutations. Subsequently accumulated mutations, SNVs and CNVs, are assumed to be neutral^66^,^67^. Variants are acquired randomly across all chromosome pairs such that no two copy number events overlap along the same lineage. SNVs and CNVs may overlap, in which case the timing of the events is used to determine the allele fraction of SNVs at the affected locus. CNV length is sampled from the observed length distribution in ref. 68. After *m* spatially distinct metastases reached the detection size *M*=10^8^, the simulation is stopped. Note that new metastases can also be seeded from previously seeded metastases.

To model the biopsy and sequencing process, a single sample consisting of one million cells of each of the *m* metastases consistent to the considered purity (15%, 35%, 55%, 75%, 95%) is subject to *in silico* sequencing. Metastases with a mixture of ancestries (polyphyletic samples) are simulated by random sampling from two distinct sites proportional to the tumour sizes at these sites (size of the second site possibly below the detection limit). Sequencing depth is negative-binomially distributed with a given mean (25, 50, 100, 200, 400, 800). A sequencing error rate of *e*=0.5% is assumed. The simulation output is the number of variant and reference ‘reads' in each metastasis sample for each mutated locus present with a VAF of at least 5% and supported by at least four variant reads (two in the case of a coverage of 25) in any of the sampled metastases. An example for a simulated phylogeny is depicted in Fig. 3a. Simulated phylogenies are available on github: https://github.com/johannesreiter/treeomics.

We compared Treeomics to standard phylogenetic reconstruction (maximum parsimony^69^, neighbour joining^69^) and modern tumour phylogeny reconstruction methods (LICHeE^37^, PhyloWGS^36^). Two different error metrics demonstrate the performance of Treeomics against existing methods: branching error and mutation matrix error score. The branching error quantifies the accuracy of the reconstructed coalescent relationships among distinct sites. From the true coalescent tree among metastatic sites, the collection of coalescent events among the sites is computed and compared with those predicted by the method. The branching error is defined as the fraction of true coalescent events missed by the reconstruction method. Since maximum parsimony and neighbour joining trees do not infer the evolutionary relationships among individual variants, the branching error metric was used to compare these methods (Fig. 3).

The mutation matrix error score quantifies the accuracy of the reconstructed sequence of mutations acquired during an evolutionary process. For a tumour with *k* parsimony-informative mutations across *m* metastases, a *k* by *k* matrix *A* is constructed where *A*_*i,j*_=1 if mutation *i* is parental to mutation *j* and 0 otherwise. If two mutations are acquired on the same branch in the true phylogeny, the correct evolutionary ordering among this pair of mutations is not required and *A*_*i,j*_=0.5. In PhyloWGS, where many phylogenies are sampled, this reconstructed phylogeny mutation matrix 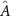 is averaged over all samples. If a tool did not provide any information about a pair of mutations *i*,*j*, 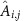 is set to *A*_*i,j*_−0.5. For the reconstructed matrix 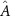, the normalized error score is computed as 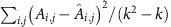. Because LICHeE and PhyloWGS do not directly infer the coalescent relationship among sites, the mutation matrix error score was used in the benchmarking (Fig. 4, Supplementary Figs 11 and 12). Recall that only founder and parsimony-informative mutations were provided as input to PhyloWGS while LICHeE and Treeomics also had to deal with noisy private mutations. PhyloWGS was run with 2,500 MCMC iterations and 5,000 inner Metropolis-Hastings iterations for a maximum of 15 h for each individual case. Increasing the number of samples and iterations did not significantly decrease the mutation matrix error score. LICHeE was run with the default parameter values except that we set *maxVAFAbsent* and *minVAFPresent* to 0.05 as well as *minClusterSize* and *minProfileSupport* to 1. These parameter changes significantly improved the performance of LICHeE in our data set.

### Binary present/absent classification

We perform conventional binary present/absent classification of each variant to allow a comparison to the inferred classification used in our new approach. We scored each variant by calculating a *P* value in all samples (one-tailed binomial test): 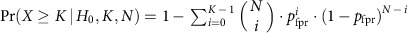 where *N* denotes the coverage, *K* denotes the number of variant reads observed at this position, and *X* denotes the random number of false-positives. As null hypothesis *H*_0_, we assume that the variant is absent. Similar to Gundem *et al*.^21^, we assumed a false-positive rate (*p*_fpr_) of 0.5% for the Illumina chip-based targeted deep sequencing. We used the step-up method^70^ to control for an average false-discovery rate of 5% in the combined set of *P* values from all samples of a patient. Variants with a rejected null hypothesis were classified as present. The remaining variants were classified as absent.

### Code availability

The source code and a manual for Treeomics, as well as multiple examples illustrating its usage, are provided at https://github.com/johannesreiter/treeomics as well as in Supplementary Software. Treeomics v1.5.2 was used for the entire analysis. The tool is implemented in Python 3.4. The inputs to the tool are the called variants and the corresponding sequencing data, either in tab-separated-values format or as matched tumour-normal VCF files. As output, Treeomics produces a comprehensive HTML report (see github repository) including statistical analysis of the data, a mutation table plot and a list of putative artifacts (false-positives, well-powered and under-powered false-negatives). Additionally, Treeomics produces evolutionary trees in LaTeX/TikZ format for high-resolution plots in PDF format. If circos is installed, Treeomics automatically creates the evolutionary conflict graph and adds it to the HTML report. Treeomics also supports various filtering (for example, minimal sample median coverage, false-positive rate, false-discovery rate) for an extensive analysis of the sequencing data. Detailed instructions for the filtering and analysis are provided in the readme file in the online repository. For solving the MILP, Treeomics makes use of the common CPLEX solver (v12.6) from IBM.

### Data availability

Targeted sequencing data of subjects Pam01, Pam02, and Pam03 have been deposited in the github repository in the directory /src/input/Makohon2016 and are also provided in Supplementary Data 1–3. All other relevant data are available within the article and its Supplementary Files or available from the corresponding authors.

## Additional information

**How to cite this article:** Reiter, J. G. *et al*. Reconstructing metastatic seeding patterns of human cancers. *Nat. Commun.* **8,** 14114 doi: 10.1038/ncomms14114 (2017).

**Publisher's note:** Springer Nature remains neutral with regard to jurisdictional claims in published maps and institutional affiliations.

## Supplementary Material

Supplementary Information Supplementary Figures, Supplementary Tables, Supplementary Methods and Supplementary References

Supplementary Data 1 Deep targeted sequending data of subject Pam03. Number of reads reporting the variant.

Supplementary Data 2 Deep targeted sequencing data of subject Pam01. Number of reads reporting the variant.

Supplementary Data 3 Deep targeted sequencing data of subject Pam02. Number of reads covering the site of the variant.

Supplementary Software 1 Treeomics v1.5.2
