# Supplementary material for: Reconstructing metastatic seeding patterns of human cancers
Source: Nat Commun. 2017 Jan 31;8:14114. doi: 10.1038/ncomms14114 (PMC5290319; doi:10.1038/ncomms14114)
Supplement: Supplementary Information — Supplementary Figures, Supplementary Tables, Supplementary Methods and Supplementary References [file ncomms14114-s1.pdf]

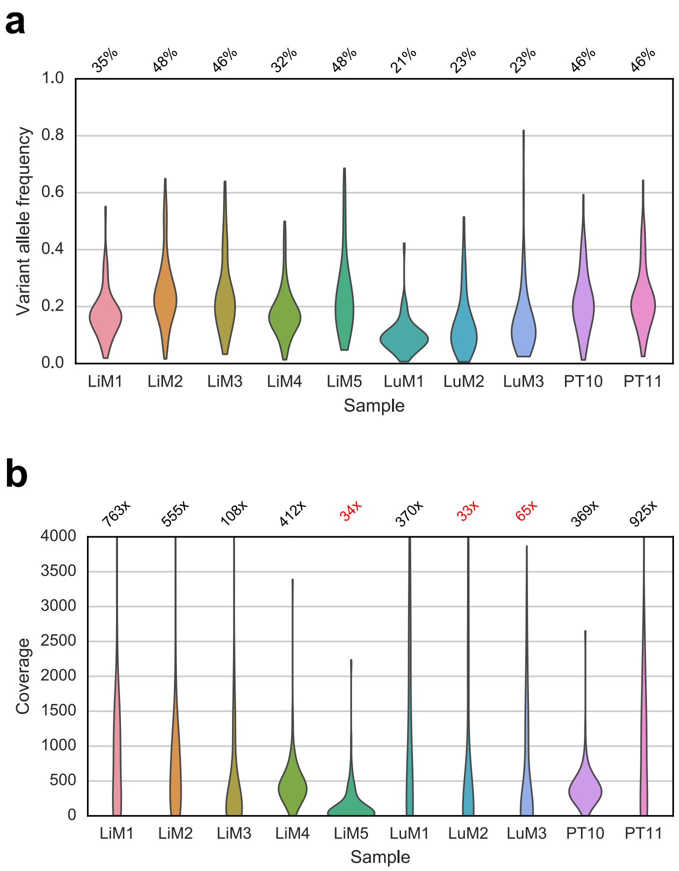

Supplementary Figure 1: Targeted sequencing data of ten samples from pancreatic cancer patient Pam03[1,2]. a | Violin plots depicting VAF distribution in each sample. Numbers at the top denote the by *Treeomics* estimated neoplastic cell content in each sample. **b** | Violin plots depicting the sequencing depth distribution across variants in each sample. Numbers at the top denote the median sequencing depth in each sample. Pancreatic ductal adenocarcinoma: LiM 1-5 (liver metastases), LuM 1-3 (lung metastases), PT 10 and 11 (primary tumor). Sequencing data are provided in Supplementary Data 1.

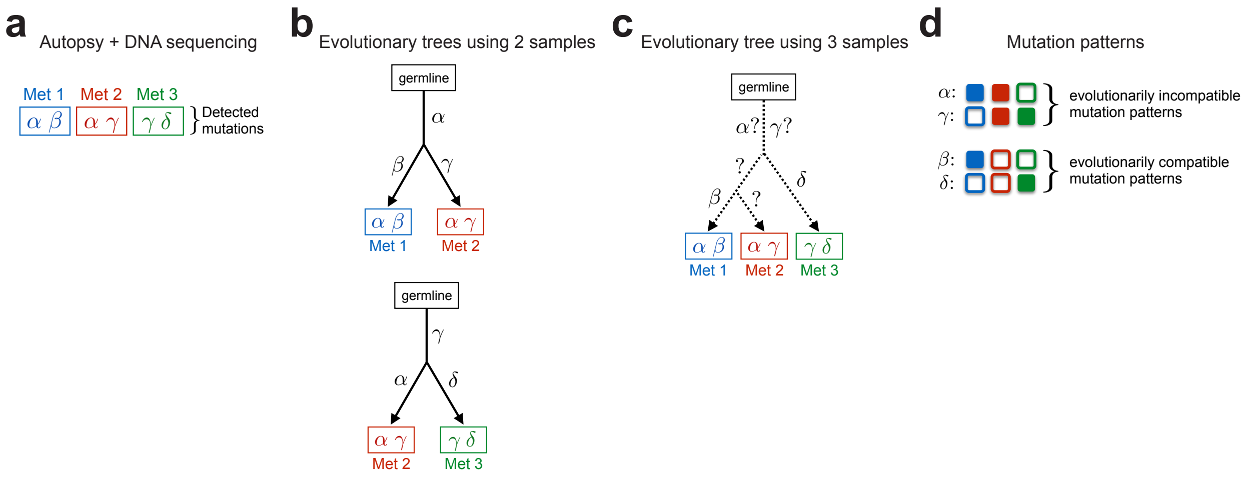

Supplementary Figure 2: Illustrative reconstruction of the evolutionary history from noisy DNA sequencing data. a | DNA sequencing is performed on samples from three spatially-distinct metastases. Variants α and β are detected in sample of Met 1 (blue), α and γ in sample of Met 2 (red), and γ and δ in sample of Met 3 (green). **b** | Analyzing samples of Met 1 and Met 2 together suggests that α was acquired before γ. Analyzing samples of Met 2 and Met 3 together suggests that γ was acquired before α. **c** | If all three samples are analyzed together, α and γ create an evolutionary contradiciton. **d** | Variants α and γ constitute evolutionarily incompatible mutation patterns since there are samples where both variants are present and samples where either of the two variants are present and the other one is absent. No perfect and persistent phylogeny exists for the given data.

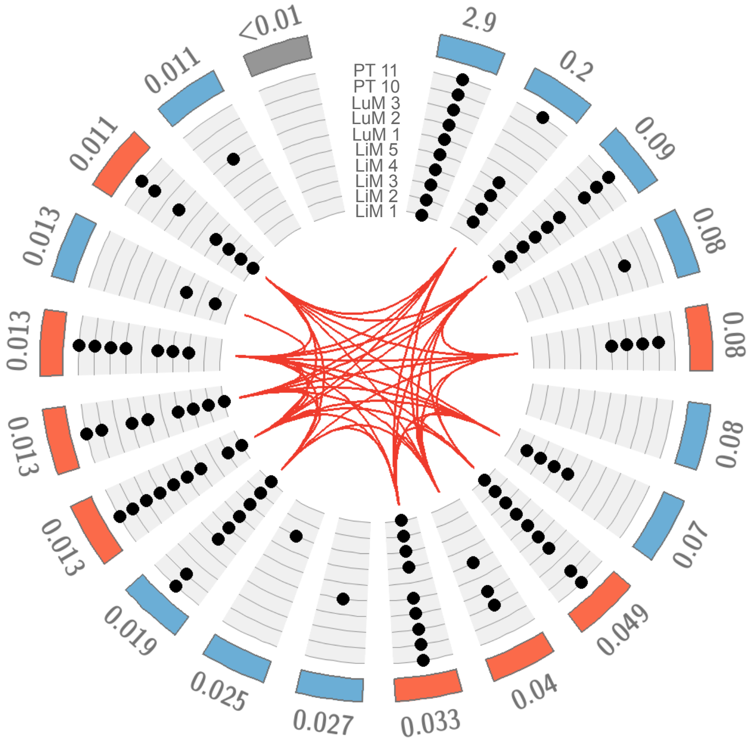

Supplementary Figure 3: Evolutionary conflict graph of the sequencing data of patient Pam03. Evolutionary incompatibilities are demarcated by red lines (edges) in the center of the circle and connect each pair of evolutionarily incompatible nodes (mutation patterns). Each sample is depicted by a circular line. Black dotes denote the presence of variants in the corresponding sample. Numbers denote the reliability score of each mutation pattern. Blue colored nodes were identified as evolutionarily compatible and red colored nodes were identified as evolutionarily incompatible by *Treeomics*. Nodes with a reliability score below 0.01 are not shown. Pancreatic ductal adenocarcinoma: LiM 1-5 (liver metastases), LuM 1-3 (lung metastases), PT 10 and 11 (primary tumor).

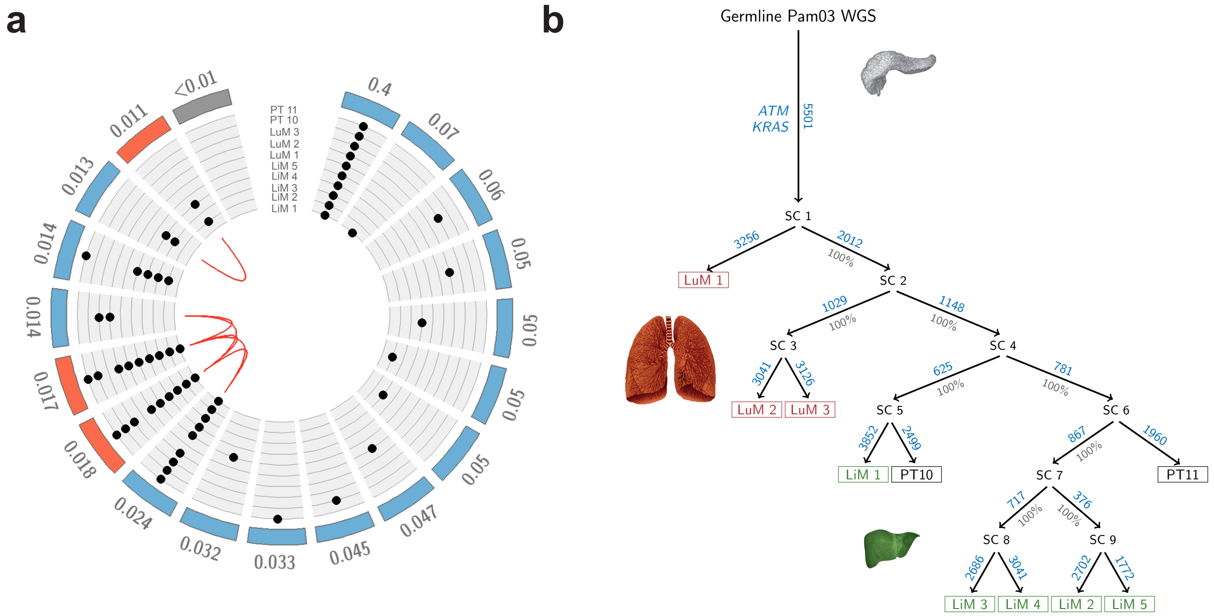

Supplementary Figure 4: Reconstructed evolution of patient Pam03's pancreatic cancer from whole genome sequencing data. a | Evolutionary conflict graph. Evolutionary incompatibilities are demarcated by red lines (edges) in the center of the circle and connect each pair of incompatible nodes (mutation patterns). Each sample is depicted by a circular line. Black dotes denote the presence of variants in the corresponding sample. Numbers denote the reliability score of each mutation pattern. Blue colored nodes were identified as evolutionarily compatible and red colored nodes were identified as evolutionarily incompatible by *Treeomics*. Nodes with a reliability score below 0.01 are not shown. **b** | Based on the identified evolutionarily compatible mutation patterns in panel **a**, a unique evolutionary tree exists. *Treeomics* reconstructed the same evolutionarily related group of samples as from targeted sequencing data (Fig. 2c). SC indicate predicted subclones. Note that *Treeomics* could not use its full statistical power because the VCF-files only provided the sequencing data of the called variants in each particular sample. Therefore, the divergence of the low-purity sample LuM 1 remained inconclusive despite the high bootstrapping values from 1000 samples (significantly fewer variants were identified by the variant caller in LuM 1). Lung metastases (LuM 1-3) are depicted in red; Liver metastases (LiM 1-5) are depicted in green; Primary tumor samples (PT 10-11) are depicted in black.

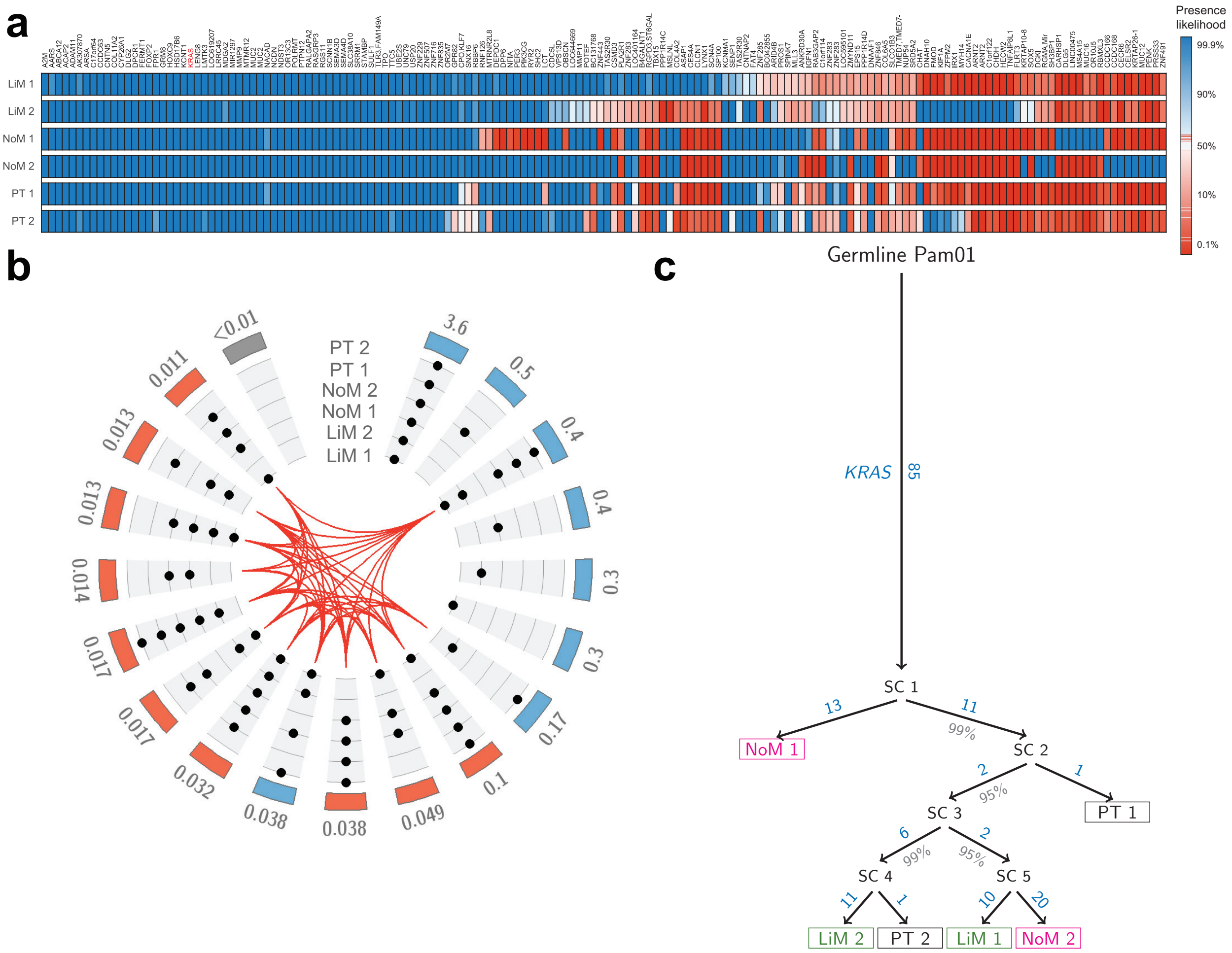

Supplementary Figure 5: Reconstructed evolutionary history of patient Pam01's pancreatic cancer[1,2]. a | Presence likelihoods of variants across six samples. Dark blue rectangles correspond to likely present variants, dark red to likely absent variants, and white to unknown presence or absence. The Bayesian classification is based on the number of supporting variant reads, the coverage, the estimated sample purity and the sequencing error rate $e = 0.5\%$. **b** | Evolutionary conflict graph. Evolutionary incompatibilities are demarcated by red lines (edges) in the center of the circle and connect each pair of incompatible nodes (mutation patterns). Each sample is depicted by a circular line. Black dotes denote the presence of variants in the corresponding sample. Numbers denote the reliability score of each mutation pattern. Blue colored nodes were identified as evolutionarily compatible and red colored nodes were identified as evolutionarily incompatible by *Treeomics*. Nodes with a reliability score below 0.01 are not shown. **c** | Evolutionary tree was reconstructed based on the identified evolutionarily compatible mutation patterns in panel **b**. Blue gene names correspond to acquired mutations in driver genes. Percentages (gray) denote bootstrap values (1,000 samples). Pancreatic ductal adenocarcinoma: liver metastases (depicted in green: LiM 1 and LiM 2), lymph node metastases (depicted in magenta: NoM 1 and NoM 2), primary tumor (depicted in black: PT 1 and PT 2). Targeted sequencing data are provided in Supplementary Data 2.

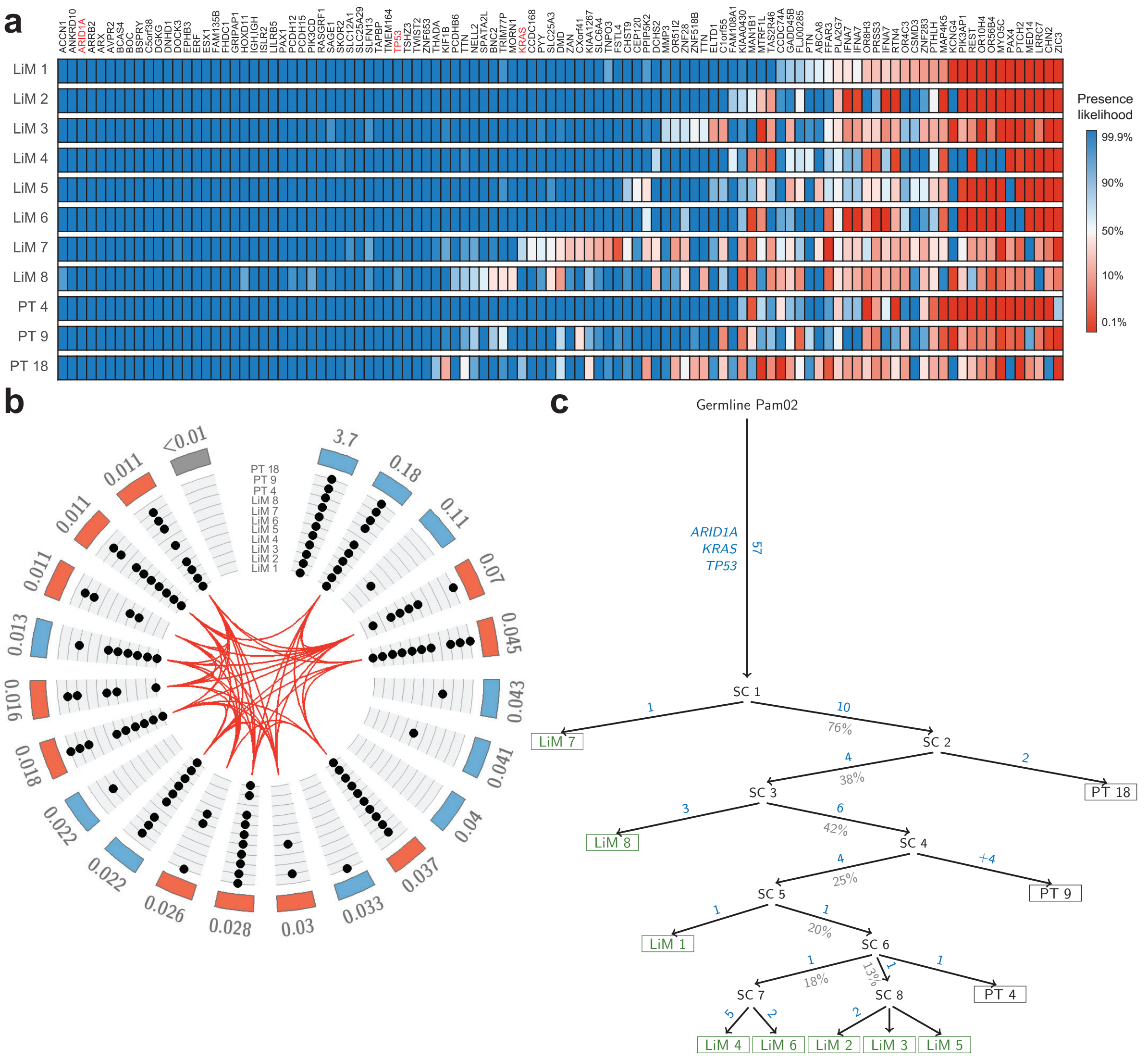

Supplementary Figure 6: Reconstructed evolutionary history of patient Pam02's pancreatic cancer[1,2]. **a** | Presence likelihoods of variants across eleven samples. Dark blue rectangles correspond to likely present variants, dark red to likely absent variants, and white to unknown presence or absence. The Bayesian classification is based on the number of supporting variant reads, the coverage, the estimated sample purity and the sequencing error rate $e = 0.5\%$ (Online Methods). **b** | Evolutionary conflict graph. Evolutionary incompatibilities are demarcated by red lines (edges) in the center of the circle and connect each pair of incompatible nodes (mutation patterns). Each sample is depicted by a circular line. Black dotes denote the presence of variants in the corresponding sample. Numbers denote the reliability score of each mutation pattern. Blue colored nodes were identified as evolutionarily compatible and red colored nodes were identified as evolutionarily incompatible by *Treeomics*. Nodes with a reliability score below 0.01 are not shown. **c** | Evolutionary tree was reconstructed based on the identified evolutionarily compatible mutation patterns in panel **b**. Blue gene names correspond to acquired mutations in driver genes. Percentages (gray) denote bootstrap values (1,000 samples). Pancreatic ductal adenocarcinoma: liver metastases (depicted in green: LiM 1-8), primary tumor (depicted in black: PT 4, 9 and 18). Targeted sequencing data are provided in Supplementary Data 3.

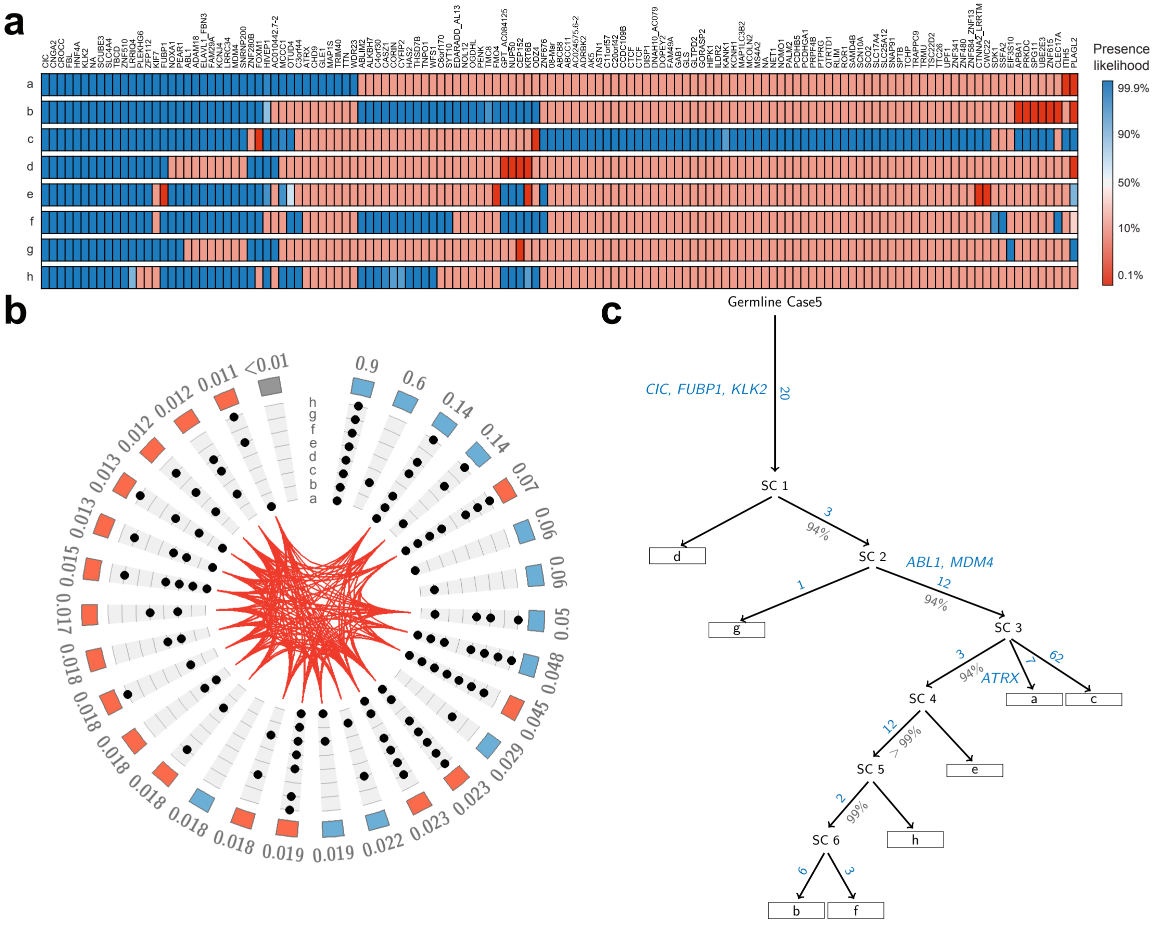

Supplementary Figure 7: Reconstructed evolution of the high-grade serous ovarian cancer of Case 5 in Bashashati et al.[3]. **a** | Presence likelihoods of deeply sequenced variants across eight samples provided in Bashashati et al. (2013, Supplementary Table 2). Dark blue rectangles correspond to likely present variants, dark red to likely absent variants, and white to unknown presence or absence. **b** | Evolutionary conflict graph. Evolutionary incompatibilities are demarcated by red lines (edges) in the center of the circle and connect each pair of incompatible nodes (mutation patterns). Black dotes denote the presence of variants in the corresponding sample. Numbers denote the reliability score of each mutation pattern. Blue colored nodes were identified as evolutionarily compatible and red colored nodes were identified as evolutionarily incompatible by *Treeomics*. Nodes with a reliability score below 0.01 are not shown. **c** | Evolutionary tree was reconstructed based on the identified evolutionarily compatible mutation patterns in panel **b**. In contrast to Bashashati et al. who suggested an early divergence of sample c, *Treeomics* clustered sample c together with sample a (other clusters are similar to their results, e.g. samples b, f, and h). Careful analysis of the sequencing data revealed that samples a and c should be clustered together as otherwise multiple validated variants would have occurred independently twice (Fig. 1D in Bashashati et al., 2013). Numbers in blue correspond to the acquired variants in the branches. Blue gene names correspond to acquired mutations in driver genes. Percentages (gray) denote bootstrap values (1,000 samples). Sample origin: right ovary (a), left ovary (b-e), left para-aortic lymph node (f), left iliac lymph node (g), left fallopian tube lesion (h). Median coverage: 12,297.5. Parameter values: error rate $e = 1\%$.

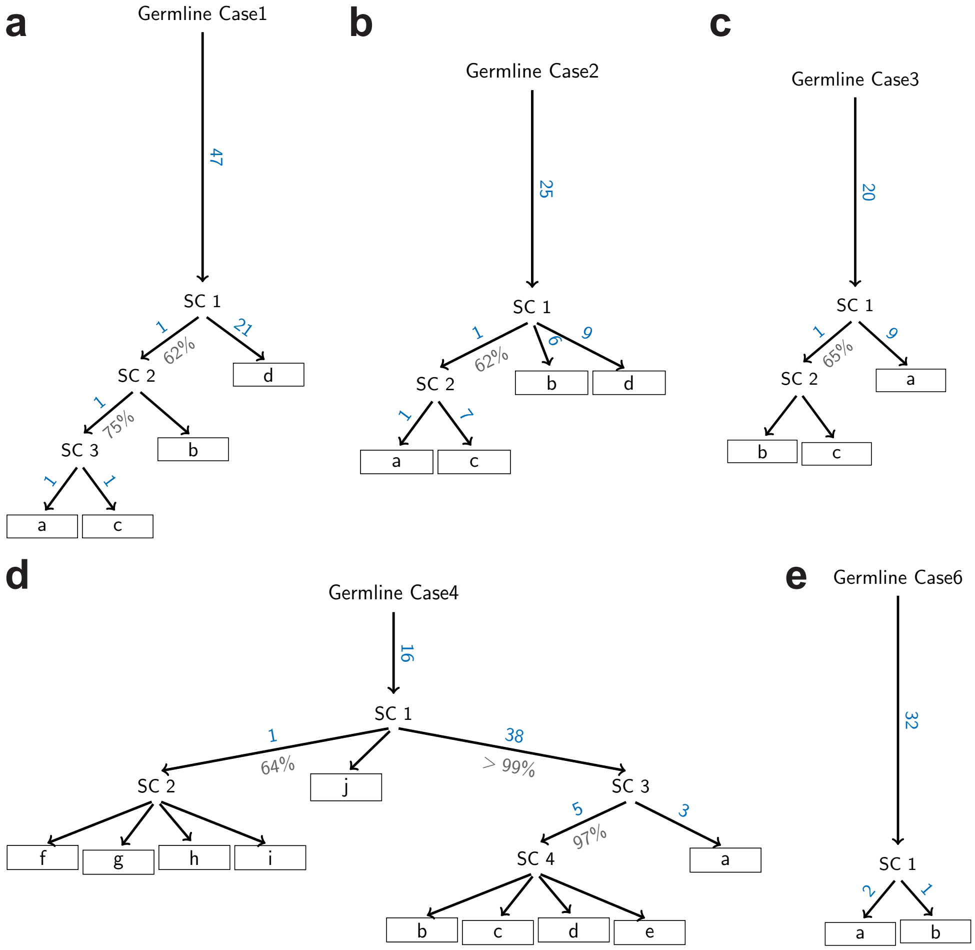

Supplementary Figure 8: Reconstructed evolution of the high-grade serous ovarian cancers in Bashashati et al.[3]. Percentages (gray) denote bootstrap values (1,000 samples). Inferred trees were identical to the ones in the original publication, except of Case 1. The low bootstrap values for Case 1 indicate the uncertainty of the by Treeomics inferred phylogyeny. Popic et al.[4] inferred yet another phylogeny for Case 1 from ours and the one reported in Bashashati et al.[3]. The different phylogenies inferred for Case 1 highlight that phylogenies without confidence measures can be very hard to interpret. Parameter values: error rate $e = 1\%$.

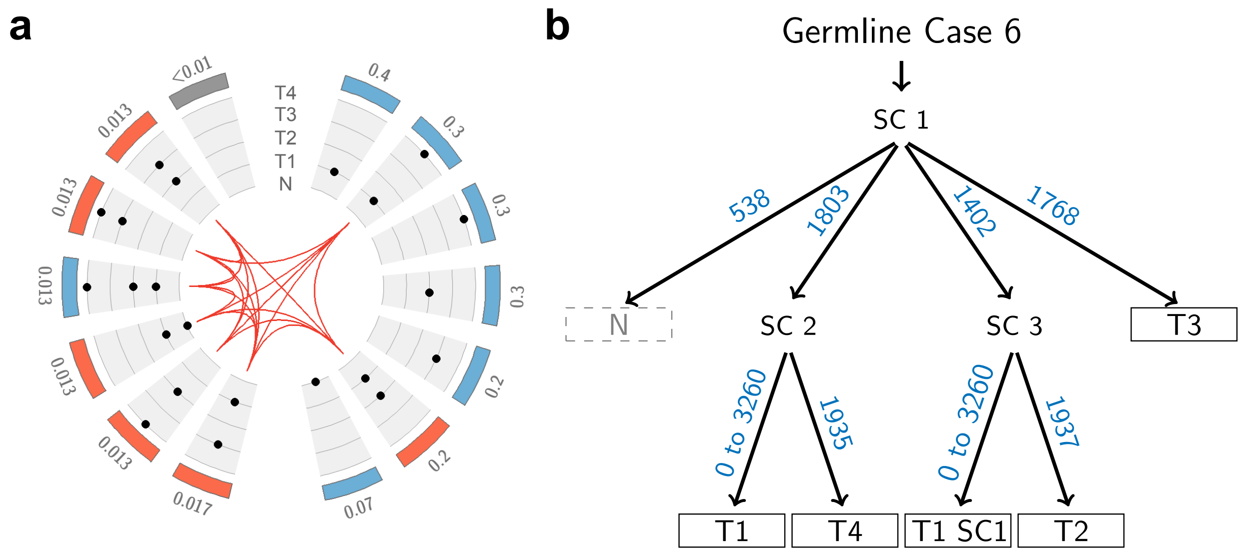

Supplementary Figure 9: Detecting putative subclones from evolutionarily incompatible mutation patterns and the evolution of the prostate cancer of case 6 in Cooper et al.[5]. a | Evolutionary conflict graph. Genome-wide DNA sequencing data was extracted from Cooper et al. (2015, Supplementary Tables 3 and 4). Each sample is depicted by a circular line. Black dotes denote the presence of variants in the corresponding sample. Evolutionary incompatibilities are demarcated by red lines (edges) in the center of the circle and connect each pair of incompatible nodes (mutation patterns). Numbers denote the reliability score of each mutation pattern. Blue colored nodes were identified as evolutionarily compatible and red colored nodes were identified as evolutionarily incompatible by *Treeomics*. Observe the high reliability score (0.2) of the evolutionarily incompatible mutation pattern to the right. Reliability scores of remaining incompatible patterns are 10-fold lower. Nodes with a reliability score below 0.01 are not shown. **b** | *Treeomics* correctly identified a subclone in sample T1 and inferred the identical evolutionary tree as in Cooper et al. (2015, Fig. 2a). SC indicate predicted subclones. Low $VAFs$ (median: 17.3%) of all variants present only in sample T1 provide additional evidence for mixed subclones in this sample. Variants detected only in sample T1 can not be assigned to one of the putative subclones by *Treeomics*. Existing methods to reconstruct the subclonal composition would assign these private mutation also to the inferred subclones[6–11]. Sample origin: spatially-distinct prostate regions. Cancerous samples are depicted in black and the normal sample is depicted in gray (dashed box).

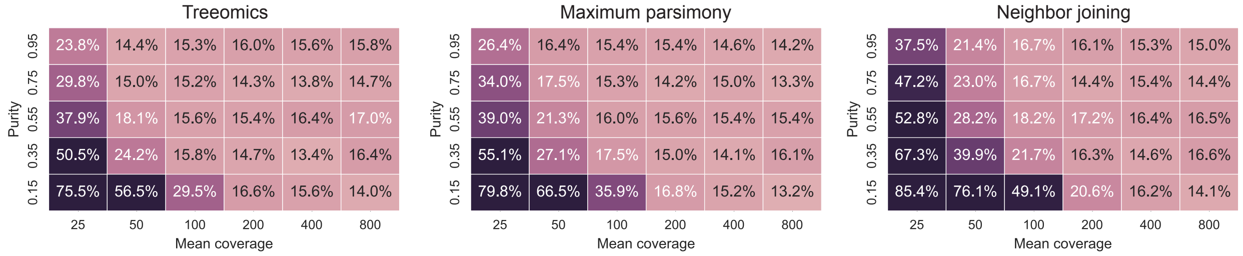

Supplementary Figure 10: Average branching error comparison between Treeomics, maximum parsimony and neighbor joining without conditioning on at least one acquired mutation per branch. Note that the branching error of ≈ 15% is due to no exonic mutations on the inner branches and therefore no tool can correctly reconstruct these branching patterns based on whole exome sequencing data alone. In the case of an elevated mutation rate, this lower bound on the branching error can further decrease. For each combination of sample purity and mean coverage, 500 independently simulated phylogenies were considered. See Online Methods for branching error calculation. Necessary binary present/absent classification for maximum parsimony and neighbor joining was based on Treeomics Bayesian inference model (variant was present if $p > 50\%$). Simulation parameter values: number of monophyletic metastases $m = 6$, sequencing error $e = 0.5\%$, point mutation rate $u = 5 \cdot 10^{-10}$, CNV rate 0.1%.

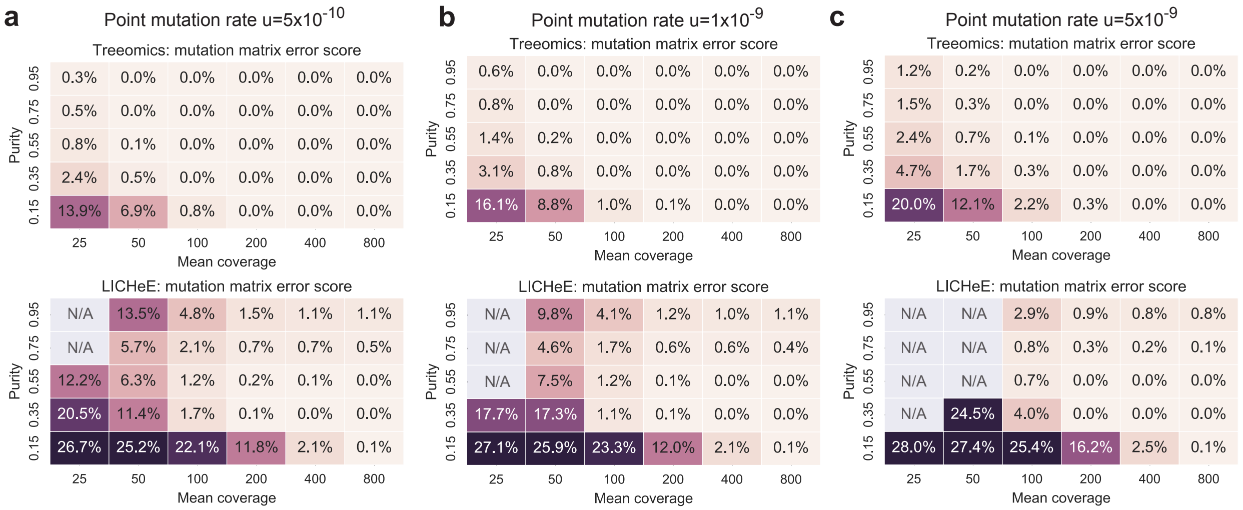

Supplementary Figure 11: Average mutation matrix error score comparison between Treeomics and LICHeE[4]. For each combination of sample purity and mean coverage, 500 independently simulated phylogenies were considered. See Online Methods for mutation matrix error score calculation. We were unable to run PhyloWGS[10] on these datasets as its runtime increased significantly with the higher number of detected variants (despite only providing founder and parsimony-informative variants). **a** | Point mutation rate of $u = 5 \cdot 10^{-1012}$. **b** | Elevated point mutation rate of $u = 10^{-9}$. **c** | Elevated point mutation rate of $u = 5 \cdot 10^{-9}$. Simulation parameter values: number of monophyletic metastases $m = 6$, sequencing error $e = 0.5\%$, CNV rate 0.1%.

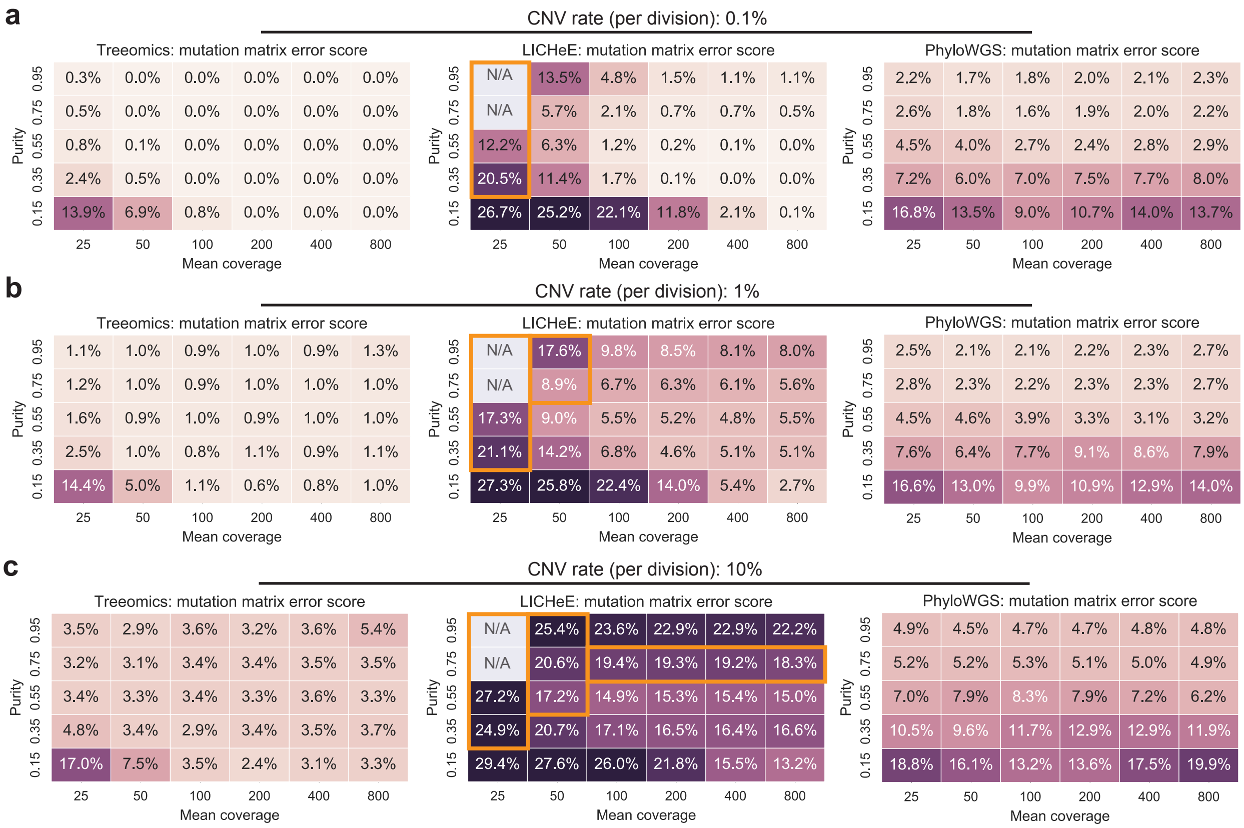

Supplementary Figure 12: Average mutation matrix error score comparison for highly chromosomally unstable cancers between Treeomics, LICHeE[4] and PhyloWGS[10]. The performance of all tools decreased with an increasing CNV accumulation rate. However, even for highly chromosomally unstable cancers *Treeomics* performs well across most scenarios[13,14]. For each combination of sample purity and mean coverage, 500 independently simulated phylogenies were considered. See Online Methods for mutation matrix error score calculation. We explored a similar parameter range for the per division rate for chromosomal alterations as by Gao et al.[15]. In the orange-framed scenarios, LICHeE was unable to infer a valid folution for more than 50% of the cases. **a** | Per division CNV rate of 0.1%. **b** | Elevated CNV rate of 1%. **c** | Elevated CNV rate of 10%. Simulation parameter values: number of monophyletic metastases $m = 6$, sequencing error $e = 0.5\%$, point mutation rate $u = 5 \cdot 10^{-10}$.

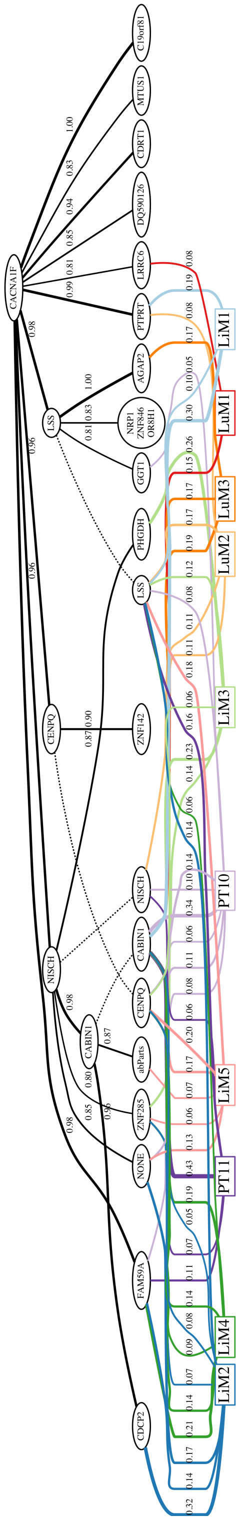

Supplementary Figure 13: Inferred subclonal sample compositions for Pam03 by AncesTree[8]. AncesTree similarily identified the evolutionarily related group of samples (LiM 2-5 and PT 11). 70% (63/90) of the variants had to be ignored such that ancestral relationships could be inferred. Used default parameter values of AncesTree: $\alpha = 0.3$, $\beta = 0.8$, $\gamma = 0.01$.

Gene name	Position	CRGalign36 within 50b	within 500b	LiM 1	LiM 2	LiM 3	LiM 4	LiM 5	LuM 1	LuM 2	LuM 3	PT 10	PT 11
ANKRD30A	chr10:37438758	Low		0.202	0.223	0.146	0.151	0.180	0.000	0.000	0.000	0.157	0.137
ATM	chr11:108192148	Normal	Low	0.224	0.272	0.197	0.241	0.157	0.104	0.188	0.179	0.229	0.281
AVEN	chr15:34331031	Normal	Normal	0.000	0.261	0.000	0.000	0.231	0.000	0.000	0.000	0.000	0.131
CALCRL	chr2:188293620	Nearby low		0.167	0.000	0.308	0.000	0.109	0.000	0.000	0.000	0.000	0.244
CDKAL1	chr6:21089124	Low		0.209	0.156	0.217	0.250	0.281	0.000	0.000	0.000	0.293	0.351
CENPQ	chr6:49456158	Normal	Low edge	0.121	0.187	0.238	0.000	0.147	0.000	0.000	0.113	0.194	0.182
DNHD1	chr11:6567175	Normal	Normal	0.113	0.273	0.273	0.244	0.217	0.167	0.000	0.123	0.200	0.233
DOCK2	chr5:169135251	Normal	Low edge	0.110	0.281	0.351	0.170	0.303	0.120	0.130	0.134	0.273	0.270
ELAVL4	chr1:50610767	Normal	Normal	0.152	0.269	0.220	0.200	0.204	0.000	0.000	0.000	0.192	0.200
FAM59A	chr18:29992995	Low		0.000	0.000	0.000	0.154	0.000	0.000	0.000	0.000	0.000	0.000
HOXD1	chr2:176972815	Normal	Normal	0.000	0.000	0.000	0.000	0.195	0.000	0.231	0.109	0.000	0.355
HTT	chr4:3156068	Normal	Low	0.216	0.291	0.214	0.173	0.260	0.124	0.200	0.136	0.310	0.189
KCTD16	chr5:143853421	Normal	Normal	0.154	0.298	0.178	0.167	0.143	0.000	0.000	0.121	0.164	0.211
KIAA1804	chr1:233497916	Normal	Normal	0.000	0.224	0.149	0.000	0.217	0.000	0.000	0.000	0.118	0.160
KRAS	chr12:25380275	Low		0.330	0.458	0.363	0.370	0.375	0.175	0.189	0.187	0.462	0.297
MNAT1	chr14:61285437	Normal	Normal	0.144	0.250	0.269	0.194	0.222	0.000	0.138	0.157	0.257	0.233
NBEAL1	chr2:203927006	Normal	Normal	0.200	0.213	0.259	0.175	0.167	0.113	0.182	0.127	0.238	0.306
NEK	chr14:75563830	Normal	Low	0.158	0.254	0.268	0.130	0.234	0.065	0.083	0.156	0.300	0.247
NRP	chr10:33559603	Normal	Low	0.000	0.000	0.000	0.000	0.000	0.000	0.106	0.011	0.000	0.000
PAIP1	chr5:43536951	Low		0.139	0.279	0.189	0.125	0.195	0.000	0.125	0.000	0.274	0.256
PIWIL4	chr11:94310464	Normal	Low edge	0.129	0.295	0.311	0.185	0.171	0.000	0.000	0.000	0.198	0.158
PRPF39	chr14:45579854	Normal	Normal	0.153	0.231	0.238	0.000	0.147	0.121	0.129	0.000	0.257	0.244
RGPD1	chr2:88124772	Low		0.000	0.000	0.000	0.101	0.000	0.000	0.000	0.000	0.000	0.000
SF3B3	chr16:70604043	Normal	Low	0.182	0.281	0.241	0.120	0.170	0.182	0.130	0.000	0.244	0.214
SNX14	chr6:86282022	Normal	Low edge	0.171	0.259	0.215	0.250	0.282	0.147	0.115	0.167	0.241	0.267
SP140	chr2:231115733	Low		0.243	0.316	0.319	0.000	0.000	0.136	0.115	0.117	0.130	0.278
WDR19	chr4:39219674	Normal	Low edge	0.160	0.337	0.280	0.310	0.316	0.000	0.129	0.000	0.182	0.169
ZNF285	chr19:44892228	Low	dbSNP site	0.108	0.000	0.000	0.000	0.000	0.000	0.000	0.000	0.000	0.000
ZNF700	chr19:12059902	Low		0.176	0.261	0.292	0.101	0.278	0.000	0.000	0.000	0.148	0.229

Supplementary Table 1: Under-powered false-negatives: 89% (49/55) of the predicted under-powered false-negatives were either present in the whole-genome sequencing data or their genome region possesses a low alignability score. Numbers greater than zero denote the variant allele frequencies in the whole-genome sequencing data of the variants classified as present. Variants marked in red are predicted under-powered false-negatives (Fig. 2b). 69% (38/55) of the predicted under-powered false-negatives were indeed present in the whole-genome sequencing data. 51% (28/55) of the predicted under-powered false-negatives possess a low alignability score[16] in the UCSC genome browser[17] in the region around the variant.

Gene name	Position	CRGalign36 within 50b	within 500b	LiM 1	LiM 2	LiM 3	LiM 4	LiM 5	LuM 1	LuM 2	LuM 3	PT 10	PT 11
SUV39H1	chrX:48564780	Normal	Normal	0.000	0.000	0.000	0.000	0.000	0.000	0.261	0.290	0.000	0.000
WBSCR17	chr7:70597468	Normal	Normal	0.000	0.000	0.000	0.000	0.000	0.000	0.000	0.000	0.186	0.000

Supplementary Table 2: Powered false-negatives: 0% (0/13) of the predicted powered false-negatives were present in the whole-genome sequencing data. Numbers greater than zero denote the variant allele frequencies in the whole-genome sequencing data of the variants classified as present. Variants marked in red are predicted powered false-negatives (Fig. 2b).

Gene name	Position	CRGalign36 within 50b	within 500b	LiM 1	LiM 2	LiM 3	LiM 4	LiM 5	LuM 1	LuM 2	LuM 3	PT 10	PT 11
abParts	chr22:23243367	Low		0.000	0.000	0.000	0.000	0.000	0.000	0.242	0.000	0.000	0.000
GGT1	chr22:25016911	Low		0.000	0.000	0.000	0.000	0.000	0.000	0.125	0.177	0.000	0.000
MTUS	chr8:17581311	Normal	Low edge	0.000	0.000	0.000	0.000	0.000	0.060	0.058	0.139	0.000	0.000
PRAMEF1	chr1:12853509	Low		0.000	0.000	0.000	0.000	0.000	0.000	0.000	0.149	0.000	0.000
PTPRT	chr20:40979337	Low		0.110	0.000	0.000	0.000	0.000	0.000	0.000	0.000	0.000	0.010
UBE2E2	chr3:22423529	Low		0.000	0.000	0.118	0.000	0.000	0.000	0.000	0.000	0.000	0.000

Supplementary Table 3: False-positives: 92% (12/13) of the predicted false-positives were also absent in the whole-genome sequencing data. Numbers greater than zero denote the variant allele frequencies in the whole-genome sequencing data of the variants classified as present. Variants marked in red are predicted false-positives (Fig. 2b).

Supplementary Methods: Bayesian Inference Model

We design the reliability score function such that the optimization problem specified by the weighted minimum vertex cover results in the maximum likelihood evolutionarily compatible mutation patterns.

Observation Likelihoods

The likelihood of a variant being present or absent is given by the number of reads reporting the variant K and the sequencing coverage N. We denote the posterior probability of a variant μ occurring in sample s as $p_{\mu,s}$. A mutation pattern ν is a vector where $\nu_s = 1$ if the mutation is present in sample s and 0 otherwise. There are $2^{|S|}$ such vectors (patterns) where S is the set of all samples.

We proceed with a binomial likelihood sampling approach, but any likelihood approach will work. A prior probability distribution of allele frequencies denoted by π and a sequencing error rate denoted by e are required. This error rate can be sample and/or mutation-specific and might be higher than the actual errors of the sequencing machine due to multi-sample filtering and variant calling. Given K variant reads and N total reads at a locus, the posterior distribution of the true fraction of variant reads f is

$$P(f|N,K) = \frac{1}{Z} \cdot \binom{N}{K} \cdot [f(1-e) + (1-f)e]^K \cdot [f \cdot e + (1-f)(1-e)]^{N-K} \cdot \pi(f) \; . \quad (1)$$

where Z is a normalizing constant. A priori, there is a nonzero probability c_0 of a variant to be absent ($f = 0$) in a particular sample. The prior π is then of the form

$$\pi(f) = c_0 \cdot \delta(f) + (1-c_0) \cdot \frac{\Gamma(\alpha+\beta)}{\Gamma(\alpha)\Gamma(\beta)} \cdot [f(1-e) + (1-f)e)]^{\alpha-1} \cdot [(1-f)(1-e) + f \cdot e]^{\beta-1} \quad (2)$$

where $\delta(f)$ denotes the Dirac delta function and Γ denotes the gamma function (used to express the beta function with the shape parameters α and β). Since the posterior probability that a given variant is absent in a sample s also depends on the sample's neoplastic cell content, we set the default values of the hyperparameters to $\alpha = 1.0$ and to $\beta = 1.0/\gamma_s$ where γ_s is the estimated sample purity. The sample-specific prior enables us to account for the varying neoplastic cell content in the different samples (Fig. 14).

The posterior probability that a variant μ in sample s is present with a true fraction of

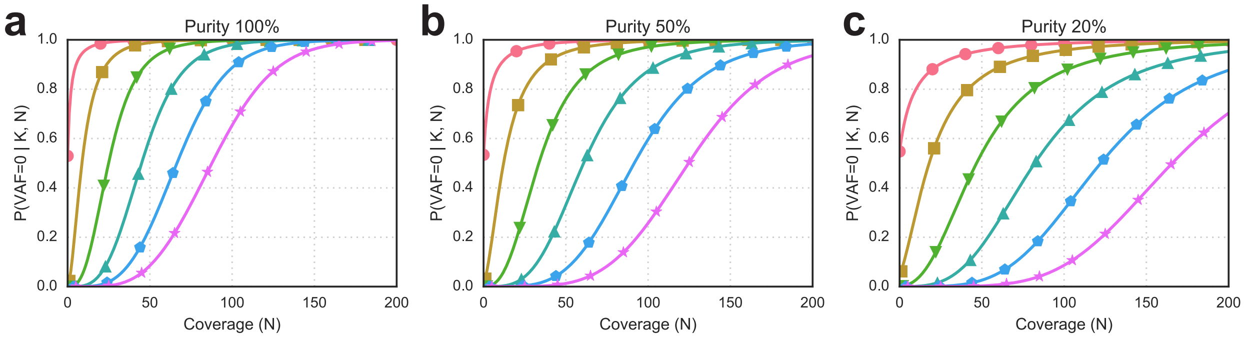

Supplementary Figure 14: Posterior probability that a variant is absent in samples with varying neoplastic cell contents. Panels **a-c** show the posterior probability that a variant reported in K sequencing reads and covered by N sequencing reads in total is absent in a sample with a given neoplastic cell content (100%, 50%, 20%). By construction of our Bayesian model, a variant with low coverage in some samples will have rather low mutation pattern likelihoods L even if the coverage is high in the remaining samples. Note that for a sample with low neoplastic cell content (e.g., panel **c**) the posterior probability that a variant is absent is lower for the same K and N than as in a sample with high neoplastic cell content (e.g., panel **a**). Parameter values: sequencing error rate $e = 1\%$, nonzero probability $c_0 = 0.5$, maximal absent frequency $f_{\text{absent}} = 0.05$.

variant reads f is

$$p_{\mu,s} = 1 - P(f \leq f_{\text{absent}} \cdot \gamma_s | N_{\mu,s}, K_{\mu,s}) \tag{3}$$

where f_{absent} is the maximal frequency threshold for an absent SNV. To ensure a smooth solution space exploration we introduce a lower and an upper bound of $p_{\mu,s}$. We assume an upper bound of one minus the probability that a variant is independently acquired twice and detected in the bulk sequencing data. We approximate the probability to acquire the same variant twice as the number of detected mutations divided by the number of sequenced base pairs ($\approx 1-2\cdot 10^{-6}$; assuming $\approx$ 1 exonic mutation per megabase[18] and 45 megabases covered by Illumina exome sequencing). The lower bound for $p_{\mu,s}$ is assumed to be $\approx 10^{-2} - 10^{-4}$ (default: 10^{-4}) to account for the possibility of loss of heterozygosity[19].

We assume a priori that mutations occur independently between samples, and that variants exhibit mutation patterns independently of other variants. The joint likelihood of a particular mutation μ having mutation pattern ν is

$$L_\mu(\nu) = \prod_{s \in S} (p_{\mu,s})^{\nu_s} \cdot (1 - p_{\mu,s})^{1-\nu_s} \ . \tag{4}$$

If we sum over all mutation patterns ν for a particular variant μ, we obtain

$$\sum_{\nu} L_{\mu}(\nu) = 1 \ . \tag{5}$$

If we fix the mutation pattern ν, then we obtain the probability that all variants exhibit ν

$$L(\nu) = \prod_{\mu} L_{\mu}(\nu) = Pr(\text{all mutations have pattern } \nu) \ . \tag{6}$$

Finally, we observe that the likelihood that no mutation (assuming that mutations are independent of each other and across samples) has pattern ν is

$$E_{\nu} = \prod_{\mu} (1 - L_{\mu}(\nu)) \ . \tag{7}$$

Purity estimation. We developed a simplistic method to estimate the purity γ_s of a sample s by taking two times the median VAF of all founding (present in all samples) and parsimony-informative mutations (present in more than one but not in all samples; preferably those present in the majority of samples) in a given sample. However, a variety of more sophisticated purity estimation methods can be used here. Despite possible aneuploidy of cancer cells, we find that the median mean absolute error of our estimator is 2% across purities from 15% to 95% and mean coverages from 25 to 800 in our simulated data sets (Supplementary Table 4). We could also use cancer cell fractions (CCFs) in the model which may further increase the accuracy of *Treeomics*. Multiple other tools are available to infer CCFs[20–22]. For some cancers (e.g., low-cellularity cancers) and some data types (e.g. targeted sequencing data[23]), CCF esimation is challenging. For generality, we therefore chose to directly use *VAFs* (possibly of SNVs in copy-number-neutral regions).

Reliability Scores

We define the reliability score (weight function) ω_{ν} of a particular mutation pattern ν (node in the evolutionary conflict graph) as

$$w_{\nu} = \frac{-\log(E_{\nu})}{m} \ . \tag{8}$$

With this weight function normalized by the number of considered variants m, the mini-

**Purity**	**Coverage** 25	50	100	200	400	800
15%	10.0%	5.0%	2.3%	1.5%	1.1%	0.9%
35%	6.3%	3.3%	2.0%	1.5%	1.1%	0.9%
55%	5.8%	3.6%	2.4%	1.8%	1.3%	0.9%
75%	5.7%	3.9%	2.7%	1.8%	1.4%	0.9%
95%	4.4%	3.4%	2.5%	1.8%	1.5%	1.0%

Supplementary Table 4: Mean absolute error of the Treeomics purity estimation across a wide range of mean sequencing depths and neoplastic cell contents. Data were simulated by a stochastic continuous-time multi-type branching process (see Online Methods for details). 500 instances per considered scenario. Parameter values: point mutation rate $u = 5 \cdot 10^{-10}$, CNV accumulation rate 0.1%.

mum weight vertex cover corresponds to the set of mutation patterns which are most likely to jointly be exhibited by no variant. This score captures the uncertainty in the sequencing data supporting a particular node. Mutation patterns supported by high quality sequencing data and multiple variants will naturally have high reliability scores w_ν. How these reliability scores are used to find the most reliable and evolutionarily compatible mutation patterns is described in Section .

TCGA code	Cancer type	Tumor samples	Mean purity	25% quantile
BLCA	Bladder Urothelial Carcinoma	101	59.5%	44%
BRCA	Breast Invasive Carcinoma	838	59.1%	46%
COAD	Colon Adenocarcinoma	400	63.8%	52%
GBM	Glioblastoma Multiforme	548	73.4%	63%
HNSC	Head and Neck Squamous Cell Carcinoma	299	50.1%	37%
KIRC	Kidney Renal Clear Cell Carcinoma	416	54.7%	44%
LUAD	Lung Adenocarcinoma	354	46.5%	33%
LUSC	Lung Squamous Cell Carcinoma	332	50.0%	34%
OV	Ovarian Carcinoma	555	76.8%	68%
READ	Rectum Adenocarcinoma	159	65.4%	55%
UCEC	Uterine Corpus Endometrial Carcinoma	396	72.5%	63%
		4398	63.3%	44%

Supplementary Table 5: Reanalyzed estimated purity in 4398 published samples across 11 cancer types. According to ABSOLUTE the mean purity across these 4398 samples from The Cancer Genome Atlas (TCGA) is 62.3%[20,24], despite the TCGA rule to only accept samples with a cellularity above 60% and therefore excluding low-cellularity cancers as pancreatic. 28% (1221) of all samples have a cellularity below 50%[24,25].

Supplementary Methods: Subclone Detection Algorithm

In this section we describe how *Treeomics* detects subclones with evolutionarily conflicting trajectories based on the identified mutation patterns and their reliability scores. If an identified evolutionarily incompatible mutation pattern has a reliability score higher than expected just by false-positives and false-negatives, we hypothesize that a metastasis might have been co-seeded by multiple sites. *Treeomics* evaluates these different scenarios. If there is sufficient evidence, *Treeomics* generates additional subclones to better explain the given data and reconstructs the evolutionary history of a subject by separating the evolutionary trajectories of the identified subclones. The following pseudocode explains the subclone detection procedure in detail:

1. Calculate the minimum number of variant reads k_{min} such that $p > 50\%$ (Equation (3)) at the median coverage N_{median} and estimated purity f_{pur} in each sample s (see purity estimation in Section).

2. False-positives: Calculate the probability to observe $k_{s_{\text{min}}}$ or more variant reads due to a sequencing error rate e in each sample s:

$$\mathbb{P}_{s_{\text{fp}}}(X \geq k_{s_{\text{min}}}) = 1 - \sum_{i=0}^{k_{s_{\text{min}}}-1} \binom{N_{\text{median}}}{i} \cdot e^i \cdot (1-e)^{N_{\text{median}}-i} \ . \tag{9}$$

3. False-negatives: Calculate the probability to observe fewer than $k_{s_{\text{min}}}$ variant reads of a clonal variant in a sample s with purity f_{pur} assuming diploid cancer cells:

$$\mathbb{P}_{s_{\text{fn}}}(X < k_{s_{\text{min}}}) = \sum_{i=0}^{k_{s_{\text{min}}}-1} \binom{N_{\text{median}}}{i} \cdot \left(\frac{f_{\text{pur}}}{2}\right)^i \cdot (1 - \frac{f_{\text{pur}}}{2})^{N_{\text{median}}-i} \ . \tag{10}$$

4. The likelihood of a mutation pattern ν induced by at least one wrong variant call is then given by $L_\nu = 1 - \prod_s (1 - \mathbb{P}_{s_{\text{fp}}} - \mathbb{P}_{s_{\text{fn}}} - p_{\text{LOH}})$ where p_{LOH} denotes the probability that a previously acquired SNV is lost due to LOH[19]. In the case of SNVs in copy-number-neutral regions of the genome, p_{LOH} can be set to zero.

5. Run through all incompatible mutation patterns ν with a reliability score (Equation (8))

$$\omega_\nu > -\frac{\log\left[(1-L_\nu)^m\right]}{m} = -\log(1-L_\nu) \tag{11}$$

where m is the number of provided variants.

If all evolutionarily incompatible mutation patterns have a reliability score lower than just expected by noise, the data do not indicate the presence of a notable subclone. End procedure. Note that samples with low coverage or low purity increase this threshold and hence influence the subclone detection ability significantly (similarly as in existing tools; Fig. 4).

If some evolutionarily incompatible mutation patterns pass the threshold, we investigate each of these patterns ν in decreasing order of their reliability score:

(a) Find highest ranked conflicting mutation pattern ξ (neighbor of ν in the conflict graph; see Online Methods) that was part of the inferred solution.

(b) Take intersection Δ of ξ and ν. Mutations that caused the evolutionary incompatiblity possibly exist in distinct subclones in the samples in Δ. These mutations were acquired on different evolutionary trajectories and thereby create the incompatiblity.

(c) If the set size of Δ is exactly one, a subclone ϕ in the sample in Δ is created. If $|\Delta| > 1$, ν might be reconsidered later - we continue with the next incompatible mutation pattern ν in step (a). Note that $|\Delta|$ is at least one since ξ is a neighbor of ν in the conflict graph and hence these patterns are evolutionarily incompatible.

(d) Edit ν to ν' such that all mutations assumed to be present in the sample in Δ are assigned to the new subclone ϕ. Hence, ν and ξ become compatible. Repeat this step for all incompatible mutation patterns that are supersets or subsets of ν.

(e) Edit all compatible mutation patterns (part of the current solution) that are supersets of ν such that all mutations are assumed to be present both in the sample in Δ and in its new subclone ϕ.

(f) Update conflict graph, rerun MILP solver and go back to step 5.

We used the above described procedure in all our benchmarkings and in case 6 (see Fig. 9) to successfully identify subclones and separate their evolutionary trajectories (Fig. 4). The benchmarking results confirm the high accuracy of *Treeomics* compared to existing methods.

Supplementary Methods: Mathematical Proofs

We prove that the decision variant of finding the most reliable and evolutionarily compatible set of variants in the perfect and persistent phylogeny problem with a known ancestral sequence is $\mathcal{NP}$*-complete*[26–28]. In fact, we show equivalence to fundamental results in computer science. Once this set of compatible variants has been identified, the evolutionary tree can be inferred in polynomial time[29]. First, we show a linear time reduction of the *weighted minimum vertex cover problem* (WMVC) to the *binary most reliable compatible variants problem* (MRCV). Second, we show a linear time reduction from the MRCV problem to the WMVC problem. Since both our reductions are *approximation preserving*, the two problems are computationally equivalent.

The WMVC problem is defined as follows: Given an undirected graph $G = (V, E)$ and a positive weight function w: $V \to \mathbb{R}^+$, find a subset of nodes $\sigma \subseteq V$ (called vertex cover) such that for all edges $(u, v) \in E$ at least one endpoint (u or v) is in the vertex cover σ and the sum $\omega = \sum_{v \in \sigma} w(v)$ is minimized (Fig. 15). The decision variant of the optimization problem asks: Does G have a vertex cover of weight at most Ω? The decision variant of the WMVC problem is $\mathcal{NP}$-complete[26,27]. A δ-approximation algorithm ($\delta > 1$) guarantees that the weight ω of the produced solution σ for any input graph G and weight function w is at most δ times larger than the weight ω^* of the optimal solution σ^*, therefore, the following inequality must hold $\omega \leq \delta \cdot \omega^*$.

The MRCV problem is defined as follows: Given an m by n binary variant matrix A describing $N = \{1, \dots n\}$ samples with $M = \{1, \dots m\}$ variants (mutations) and a reliability vector $B \in \mathbb{R}^{+m}$, find a subset of variants $S \subseteq M$ such that the sum of their reliabilities $\sum_{i \in S} B_i$ is maximized and all variants $i \in S$ are evolutionarily compatible. Two variants $k_1 \in M$ and $k_2 \in M$ are evolutionary incompatible iff (if and only if) four samples $l_1, l_2, l_3, l_4 \in N$ with the following mutation patterns exist: (i) both variants are absent ($A_{k_1,l_1} = A_{k_2,l_1} = 0$; germline sample), (ii) mutation k_1 is present and k_2 is absent ($A_{k_1,l_2} = 1$, $A_{k_2,l_2} = 0$), mutation k_1 is absent and k_2 is present ($A_{k_1,l_3} = 0$, $A_{k_2,l_3} = 1$), and both variants are present ($A_{k_1,l_4} = 1$, $A_{k_2,l_4} = 1$). The decision variant of the MRCV problem asks: Does A have a subset of compatible variants of reliability at least Ω?

1. **Reduction from the WMVC problem to the MRCV problem.** The input graph $G = (V, E)$ of the weighted minimum vertex cover problem can be directly interpreted as the conflict graph in a phylogeny problem (see Online Methods for more details). Each node $v \in V$ corresponds to exactly one variant k_v with a unique

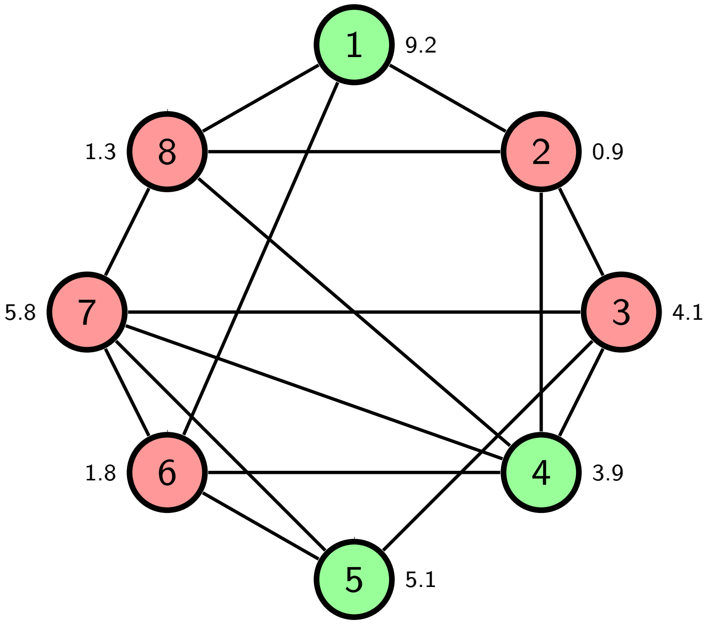

Supplementary Figure 15: Red nodes represent the minimum weight vertex cover of an undirected graph. The optimal solution $\{2, 3, 6, 7, 8\}$ covers all edges and is of weight 13.9. The complement set of the solution $\{1, 4, 5\}$ corresponds to the most reliable and compatible nodes (mutation patterns) and would form the solution in the perfect and persistent phylogeny problem with a weight of 18.2 (see Supplementary Table 6 for a reduction to an instance of the phylogeny problem).

mutation pattern. The reliability score of the variant k_v and thereby its mutation pattern is set to $B_v = w(v)$. The mutation patterns (i.e., the set of samples where a variant is present) are generated as follows. For each node $v \in V$ we generate a sample s_v where only variant k_v is present. For each edge $(v, u) \in E$ we generate a sample s_{vu} where both variants k_v and k_u are present (see Fig. 15 and Supplementary Table 6 for an example). Additionally, we generate a germline sample s_g where no variants are present.

By construction of the compatibility instance of the perfect and persistent phylogeny problem, two mutation patterns are evolutionary incompatible iff their corresponding nodes in the vertex cover problem are adjacent. Two variants k_v and k_u are evolutionary incompatible iff samples with the following patterns exist: only mutation k_v is present (sample s_v), only variant k_u is present (sample s_u), both variants are absent (germline sample s_g), and both variants are present (sample s_{vu}). Note that in each individual sample at most two variants (nodes) are present. A sample with two present variants k_v and k_u exists iff there was an edge $e = (v, u)$ in the original instance of the vertex cover problem. The number of required samples is in $\mathcal{O}(\mid V \mid + \mid E \mid)$. Each generated

mutation pattern represents precisely one node in the input graph.

Given an arbitrary weighted graph $G = (V, E)$, suppose that $\sigma^* \subseteq V$ is its minimal-weight vertex cover. By the definition of a vertex cover, at least one endpoint of each edge has to be in σ^*. Therefore, if we remove σ^* from V and the edges adjacent to the nodes in σ^*, no edges remain and the corresponding phylogeny has no evolutionary incompatibilities. Since σ^* is the minimal-weight vertex cover, the set $\{k_v \mid v \in V \setminus \sigma^*\}$ corresponds exactly to the most reliable and evolutionarily compatible set of variants. On the contrary, if σ^* is not the minimal-weight vertex cover, either an edge is not covered by σ^* and hence there will be a sample s_{uv} leading to incompatible mutation patterns of the variants k_v and k_u; or σ^* is not of minimal-weight and hence there will be another set of mutation patterns with a higher sum of their reliability scores. The solution of the WMVC problem represents the complement set of the solution of the MRCV problem. Note that the reduction maintains the exact value of the solution and hence is approximation-preserving (Lemma 1).

2. **Reduction from the MRCV problem to the WMVC problem.** Given an arbitrary binary phylogeny problem with an m by n variant matrix A and a reliability vector B. We construct the conflict graph $G = (V, E)$ exactly as described in the Online Methods. Each node $v \in V$ represents a particular mutation pattern of a unique subset of variants $R_v \subseteq M$ (where $\bigcup_{v \in V} R_v = M$). The weight of each node v is set to $w(v) = \sum_{k \in R_v} B_k$. For each pair of incompatible mutation patterns $v \in V$ and $u \in V$, there exists an edge $(v, u) \in E$.

   Suppose that $\hat{S} \subseteq M$ is the most reliable and evolutionarily compatible set of variants in this phylogeny problem with m variants and n samples. Since the evolutionary compatibility of variants is determined by their mutation patterns, we can directly focus on the patterns $\hat{R} \subseteq V$ of $\hat{S}$. Because $\hat{R}$ consists of all mutation patterns of the most reliable and compatible set of variants $\hat{S}$, all evolutionary incompatibilities must be caused by the patterns R^* of the variants in the complement set $S^* = M \setminus \hat{S}$. It follows that $R^* = V \setminus \hat{R}$ must cover all edges in the conflict graph and therefore R^* is the minimal vertex cover of G.

   In contrast, suppose that $\hat{S}$ is not the most reliable and evolutionarily compatible set of variants. The construction of the conflict graph guarantees that either $R^* = V \setminus \hat{R}$ does not cover all edges (resulting from evolutionarily incompatible patterns) or $\sum_{v \in R^*} w(v)$ is not minimal (resulting from not most reliable) and hence there must exist a different

**Mut. pat.**	**Samples**																								
(reliability)	s_1	s_2	s_3	s_4	s_5	s_6	s_7	s_8	s_{12}	s_{16}	s_{18}	s_{23}	s_{24}	s_{28}	s_{34}	s_{35}	s_{37}	s_{46}	s_{47}	s_{48}	s_{56}	s_{57}	s_{67}	s_{78}	s_g
1 (9.2)	1	0	0	0	0	0	0	0	1	1	1	0	0	0	0	0	0	0	0	0	0	0	0	0	0
2 (0.9)	0	1	0	0	0	0	0	0	1	0	0	1	1	1	0	0	0	0	0	0	0	0	0	0	0
3 (4.1)	0	0	1	0	0	0	0	0	0	0	0	1	0	0	1	1	1	0	0	0	0	0	0	0	0
4 (3.9)	0	0	0	1	0	0	0	0	0	0	0	0	1	0	1	0	0	1	1	1	0	0	0	0	0
5 (5.1)	0	0	0	0	1	0	0	0	0	0	0	0	0	0	0	1	0	0	0	0	1	1	0	0	0
6 (1.8)	0	0	0	0	0	1	0	0	0	1	0	0	0	0	0	0	0	1	0	0	1	0	1	0	0
7 (5.8)	0	0	0	0	0	0	1	0	0	0	0	0	0	0	0	0	1	0	1	0	0	1	1	1	0
8 (1.3)	0	0	0	0	0	0	0	1	0	0	1	0	0	1	0	0	0	0	0	1	0	0	0	1	0

Supplementary Table 6: Instance of the binary, most reliable phylogeny problem reduced from the graph depicted in Fig. 15. The compatible and most reliable mutation patterns are depicted in green. Only samples with two present variants can cause evolutionary incompatibility among mutation patterns. Each of these samples encodes an edge in the original vertex cover problem.

set $\tilde{R} \subseteq V$ which covers all edges and is minimal. Note that the reduction maintains the exact value of the solution and hence is approximation-preserving (Lemma 1).

Lemma 1. *The reduction from the WMVC problem to the MRCV problem (1.) and the reduction from the MRCV problem to the WMVC problem (2.) are approximation-preserving.*

Hence, finding the most reliable and compatible set of variants in the perfect and persistent phylogeny problem is computationally as hard as finding the minimal weighted vertex cover in a given graph and vice versa. A number of fundamental results have been established for the weighted vertex cover problem in computer science, and they are as follows. The decision problem is NP-complete[26,27], and the optimization problem can be solved via MILP (mixed integer linear programming) which is efficient for a large class of practical instance of the weighted vertex cover problem[30]. A related question of the optimization problem is whether any approximation can be obtained more efficiently. Bar-Yehuda and Even[31] and Gonzalez[32] demonstrated that the weighted vertex cover problem can be approximated very efficiently (in linear time) for a factor two approximation. However, lower bound results show that unless the complexity class $\mathcal{P} = \mathcal{NP}$ (which is very unlikely), no factor 1.3606 approximation is possible[33]. Moreover, under the widely believed Unique Games Conjecture (UGC), unless $\mathcal{P} = \mathcal{NP}$, no $(2 - \epsilon)$-approximation is possible[34]. As a consequence of the equivalence that we establish between the MRCV problem and the widely studied weighted vertex cover problem, we obtain all these results for the MRCV problem. On one hand our

results include positive results (such as a practical algorithm via MILP, an efficient algorithm for 2-approximation), optimal complexity results (such as $\mathcal{NP}$-completeness), as well as negative results (inefficiency of small factor approximation). Our results also imply that all previous approaches based on heuristics either in the worst case take exponential time, or they do not even achieve a factor 2-approximation. We conclude the section with a formal statement of our result.

Theorem 1. *The following assertions hold in a perfect and persistent phylogeny:*

1. *The decision version of the MRCV problem is $\mathcal{NP}$-complete.*
2. *The optimization version of the MRCV problem can be solved by MILP (mixed integer linear programming).*
3. *A 2-approximation of the optimization version of the MRCV problem can be solved in linear time.*
4. *A 1.3606-approximation of the optimization version of the MRCV problem is not possible unless $\mathcal{P} = \mathcal{NP}$.*
5. *Under the widely believed Unique Games Conjecture (UGC), for any $\epsilon > 0$, an $(2 - \epsilon)$-approximation of the optimization version of the MRCV problem is not possible unless $\mathcal{P} = \mathcal{NP}$.*

Treeomics Manual

A detailed manual is provided at https://github.com/johannesreiter/treeomics. To run *Treeomics*, open a terminal and go to the src directory of the downloaded repository. If you face any problems setting up *Treeomics* or identify potential errors in the tool, please contact us over *github* or at http://www.people.fas.harvard.edu/~reiter. Detailed installation instruction are provided in the README.md-file. If circos is installed[35], Treeomics automatically creates the evolutionary conflict graph and adds it to the HTML report (see repository for an example). The usage of *Treeomics* is python treeomics -r <var-reads table> -s <coverage table> | -v <vcf file> -O with the following parameters:

-r <var-reads table> Path to a tab-delimited text file with the number of reads reporting a variant in each sample (for example see Pam03_1-10_mutant_reads.txt in src/input/Makohon2016).

-s <coverage table> Path to a tab-delimited text file with the coverage at the position of a variant in each sample (for example see Pam03_1-10_phredcoverage.txt in src/input/Makohon2016).

-v <VCF file> Path to a Variant Call Format file reporting all called variants across all samples.

-d <VCF file directory> Path to a directory with separate VCF files for each sample.

-n <normal sample name> If the name of the matched normal sample is provided, variants significantly present in this sample will be excluded. Generally the normal sample is excluded in the evolutionary reconstruction.

-b <no bootstrapping samples> Number of bootstrapping samples to obtain confidence values in the branching.

-u Enables subclone detection (default False).

-e <sequencing error rate> Error rate in the Bayesian inference model. Default value is $e = 1\%$.

-a <max absent VAF> : Maximum VAF for an absent variant f_{absent} before considering the estimated purity (default 5%)

`-z <prior absent probability>`: Prior probability for a variant being absent. Default value is $c_0 = 0.5$.

`-p <false positive rate>`: False-positive rate for conventional binary classification. Default value is $e = 0.5\%$ (only relevant for artifact comparison).

`-i <false discovery rate>`: Targeted false-discovery rate for conventional binary classification (only relevant for artifact comparison).

`-y <min absent coverage>`: Minimum coverage for a powered absent variant (only relevant for artifact comparison).

`-o <output directory>`: Configure output directory for the results of *Treeomics*.

`-t <time limit>`: Maximum running time for CPLEX to solve the MILP (in seconds, default `None`). If not `None`, the obtained solution is no longer guaranteed to be optimal.

`-l <max No MPS>`: Maximum number of considered mutation patterns per variant (default `None`). If not `None`, the obtained solution is no longer guaranteed to be optimal.

`--no_plots` Disables generation of plots (useful for benchmarking; default `True`).

Default parameter values as well as output directory can be changed in `src\settings.py`. Moreover, the `settings.py` provides more options on annotating driver genes and configuring the plot output names. All plots, analysis and logging files, and the HTML report will be in a user-defined output directory. We provide an example HTML report (saved as PDF) generated by *Treeomics* in the root directory of the repository.

Supplementary References

1. Makohon-Moore, A. P. *et al.* Clonal evolution defines the natural history of metastatic pancreatic cancer. *Cancer Research* **75,** 4137–4137 (2015).
2. Makohon-Moore, A. P. *et al.* Limited heterogeneity of known driver gene mutations among the metastases of individual pancreatic cancer patients. *Nature Genetics* **(in press)** (2016).
3. Bashashati, A. *et al.* Distinct evolutionary trajectories of primary high-grade serous ovarian cancers revealed through spatial mutational profiling. *The Journal of Pathology* **231,** 21–34 (2013).
4. Popic, V. *et al.* Fast and scalable inference of multi-sample cancer lineages. *Genome biology* **16,** 91 (2015).
5. Cooper, C. S. *et al.* Analysis of the genetic phylogeny of multifocal prostate cancer identifies multiple independent clonal expansions in neoplastic and morphologically normal prostate tissue. *Nat Genet* **47,** 367–372 (2015).
6. Roth, A. *et al.* PyClone: statistical inference of clonal population structure in cancer. *Nature Methods* **11,** 396–401 (2014).
7. Miller, C. A. *et al.* SciClone: Inferring clonal architecture and tracking the spatial and temporal patterns of tumor evolution. *PLoS Computational Biology* **10,** e1003665 (2014).
8. El-Kebir, M., Oesper, L., Acheson-Field, H. & Raphael, B. J. Reconstruction of clonal trees and tumor composition from multi-sample sequencing data. *Bioinformatics* **31,** i62–i70 (2015).
9. Niknafs, N., Beleva-Guthrie, V., Naiman, D. Q. & Karchin, R. SubClonal Hierarchy Inference from Somatic Mutations: Automatic Reconstruction of Cancer Evolutionary Trees from Multi-region Next Generation Sequencing. *PLoS Comput Biol* **11,** e1004416 (2015).
10. Deshwar, A. G. *et al.* PhyloWGS: Reconstructing subclonal composition and evolution from whole-genome sequencing of tumors. *Genome Biology* **16,** 35 (2015).
11. Yuan, K., Sakoparnig, T., Markowetz, F. & Beerenwinkel, N. BitPhylogeny: a probabilistic framework for reconstructing intra-tumor phylogenies. *Genome biology* **16,** 36 (2015).

12. Jones, S. *et al.* Comparative lesion sequencing provides insights into tumor evolution. *Proc Natl Acad Sci USA* **105,** 4283–8 (2008).

13. Beroukhim, R. *et al.* The landscape of somatic copy-number alteration across human cancers. *Nature* **463,** 899–905 (2010).

14. Ciriello, G. *et al.* Emerging landscape of oncogenic signatures across human cancers. *Nature Genetics* **45,** 1127–1133 (2013).

15. Gao, R. *et al.* Punctuated copy number evolution and clonal stasis in triple-negative breast cancer. *Nat Genet* **advance online publication.** doi:`10.1038/ng.3641` (2016).

16. Derrien, T. *et al.* Fast computation and applications of genome mappability. *PloS one* **7,** e30377–e30377 (2012).

17. Rosenbloom, K. R. *et al.* The UCSC genome browser database: 2015 update. *Nucleic acids research* **43,** D670–D681 (2015).

18. Lawrence, M. S. *et al.* Mutational heterogeneity in cancer and the search for new cancer-associated genes. *Nature* **499,** 214–218 (2013).

19. McPherson, A. *et al.* Divergent modes of clonal spread and intraperitoneal mixing in high-grade serous ovarian cancer. *Nature Genetics* **48,** 758–767 (2016).

20. Carter, S. L. *et al.* Absolute quantification of somatic DNA alterations in human cancer. *Nature Biotechnology* **30,** 413–421 (2012).

21. Oesper, L., Mahmoody, A. & Raphael, B. J. THetA: inferring intra-tumor heterogeneity from high-throughput DNA sequencing data. *Genome Biology* **14,** R80 (2013).

22. Ha, G. *et al.* TITAN: inference of copy number architectures in clonal cell populations from tumor whole-genome sequence data. *Genome Research* **24,** 1881–1893 (2014).

23. Smith, E. N. *et al.* Biased estimates of clonal evolution and subclonal heterogeneity can arise from PCR duplicates in deep sequencing experiments. *Genome Biology* **15,** 420 (2014).

24. Aran, D., Sirota, M. & Butte, A. J. Systematic pan-cancer analysis of tumour purity. *Nature Communications* **6.** doi:`10.1038/ncomms9971` (2015).

25. TCGA. *TCGA Tissue Sample Requirements* cancergenome.nih.gov/cancersselected/biospeccriteria. Accessed in January 2016.

26. Karp, R. M. in *Complexity of Computer Computations* 85–103 (Springer US, 1972). doi:`10.1007/978-1-4684-2001-2_9`.

27. Garey, M. R. & Johnson, D. S. *Computers and Intractability: A Guide to the Theory of NP-Completeness* (WH Freeman, USA, 1979).

28. Day, W. H. & Sankoff, D. Computational complexity of inferring phylogenies by compatibility. *Systematic Biology* **35,** 224–229 (1986).

29. Gusfield, D. Efficient algorithms for inferring evolutionary trees. *Networks* **21,** 19–28 (1991).

30. Nemhauser, G. L. & Wolsey, L. A. *Integer and combinatorial optimization* (Wiley New York, 1988).

31. Bar-Yehuda, R. & Even, S. A linear-time approximation algorithm for the weighted vertex cover problem. *Journal of Algorithms* **2,** 198–203 (1981).

32. Gonzalez, T. F. A simple LP-free approximation algorithm for the minimum weight vertex cover problem. *Information Processing Letters* **54,** 129–131 (1995).

33. Dinur, I. & Safra, S. On the hardness of approximating minimum vertex cover. *Annals of Mathematics,* 439–485 (2005).

34. Khot, S. & Regev, O. Vertex cover might be hard to approximate to within $2 - \varepsilon$. *Journal of Computer and System Sciences* **74,** 335–349 (2008).

35. Krzywinski, M. *et al.* Circos: an information aesthetic for comparative genomics. *Genome research* **19,** 1639–1645 (2009).
