# Supplementary material for: Reconstructing metastatic seeding patterns of human cancers
Source: Nat Commun. 2017 Jan 31;8:14114. doi: 10.1038/ncomms14114 (PMC5290319; doi:10.1038/ncomms14114)
Supplement: Supplementary Software 1 — Treeomics v1.5.2 [file ncomms14114-s5.zip › Treeomics_v1.5.2/example_analysis_report_Pam03.pdf]

# Treeomics analysis report of patient Pam03

## Input data

| Sample | Median coverage (mean) | Median MAF (mean) | Purity | No variants present | absent |
|---|---|---|---|---|---|
| LiM 1 | 763.0 (867.8) | 16.4% (17.2%) | 35.0% | 62 | 31 |
| LiM 2 | 555.0 (717.5) | 22.1% (24.3%) | 47.8% | 74 | 19 |
| LiM 3 | 108.0 (468.8) | 22.5% (24.3%) | 46.1% | 71 | 22 |
| LiM 4 | 412.0 (474.7) | 16.7% (17.8%) | 32.4% | 73 | 20 |
| LiM 5 | 34.0 (139.2) | 21.4% (25.5%) | 48.1% | 64 | 29 |
| LuM 1 | 370.0 (1196.1) | 9.2% (10.2%) | 20.6% | 66 | 27 |
| LuM 2 | 33.0 (635.1) | 12.2% (14.1%) | 22.8% | 54 | 39 |
| LuM 3 | 65.0 (612.4) | 11.5% (15.2%) | 23.4% | 56 | 37 |
| PT 10 | 369.0 (415.2) | 20.7% (21.5%) | 46.1% | 68 | 25 |
| PT 11 | 925.0 (1037.4) | 20.8% (21.8%) | 45.5% | 64 | 29 |

Samples that passed the filtering: 10/10
Median coverage in the passed samples: 302.0 (mean: 656.43)

Total number of passed somatic variants: 93
Variants classified as present in at least one of the samples that passed the filtering: 88
Founders (variants present in all samples): 40 (45.5%)
Mean number of unique (private) variants per sample: 0.7 (1.1%)

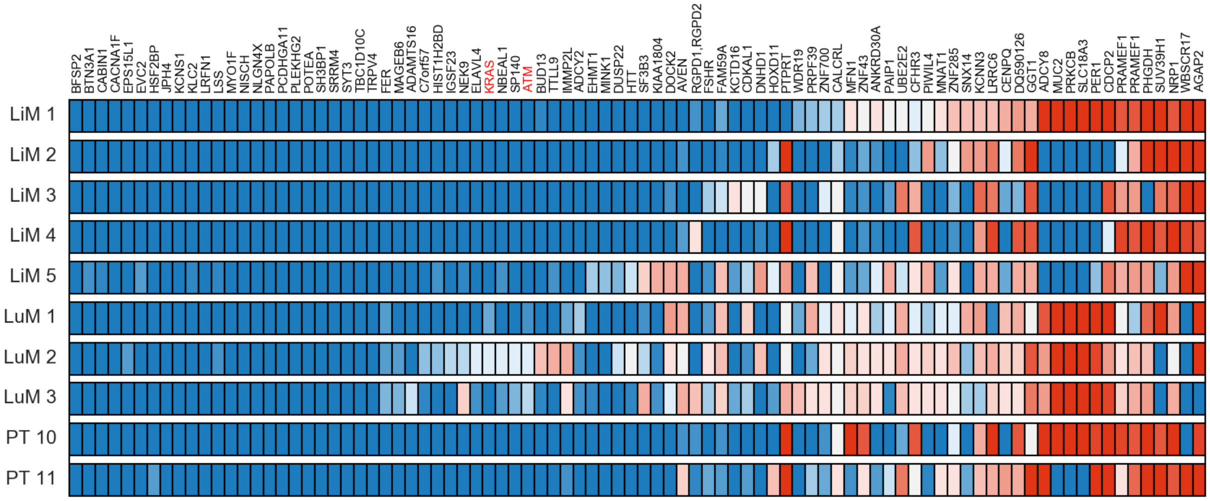


**Probabilistic variant classification across 10 samples of patient Pam03.** Blue rectangles correspond to present variants, red to absent variants, and white to unknown mutation status. Brighter colors denote higher probability.

## Evolutionary conflict graph

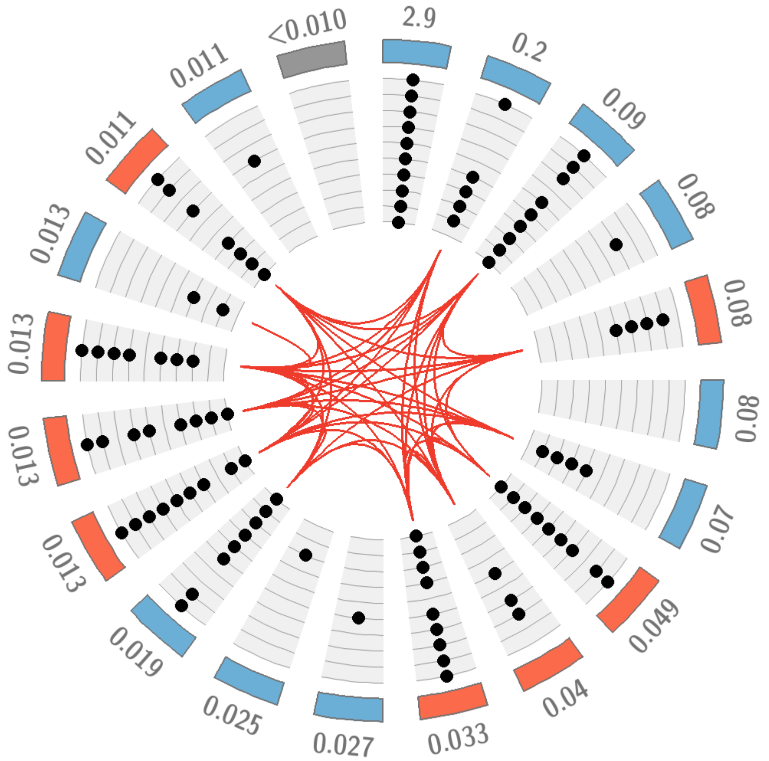


**Evolutionary conflict graph of 10 samples in patient Pam03.** Treeomics considered 1025 distinct mutation patterns (MPs). Each circular line represents a distinct sample. Inner to outer lines denote: LiM 1, LiM 2, LiM 3, LiM 4, LiM 5, LuM 1, LuM 2, LuM 3, PT 10, PT 11. Marks on these lines denote present variants. Labels denote the MP reliability scores. Only nodes with the highest reliability score are depicted. Blue colored nodes (MPs) are evolutionarily compatible and red colored nodes are evolutionarily incompatible indicated by edges among the nodes. Minimum reliability score value to be considered as a potential subclone: 0.182.

## Data artifacts

**Putative false-positives:**

- *abParts* (chr22q11.22__23243367__T>C) in samples: LiM 2 (reads: 7/555), LuM 1 (reads: 14/1139), PT 11 (reads: 13/934)
- *GGT1* (chr22q11.23__25016911__C>T) in samples: LiM 1 (reads: 17/1131), LuM 1 (reads: 32/2135), LuM 3 (reads: 14/1474)
- *MTUS1* (chr8p22__17581311__C>T) in samples: PT 11 (reads: 4/173)
- *PRAMEF1* (chr1p36.21__12853509__A>C) in samples: LiM 2 (reads: 13/674), PT 11 (reads: 14/997)
- *PTPRT* (chr20q12__40979337__G>T) in samples: LuM 1 (reads: 6/329)
- *UBE2E2* (chr3p24.3__22423529__G>C) in samples: LiM 1 (reads: 3/97), LiM 5 (reads: 2/30), PT 10 (reads: 15/279)

**Putative lost variants:**

- *SUV39H1* (chrXp11.23__48564780__T>G) in samples: LiM 1 (reads: 0/742), LiM 2 (reads: 0/513), LiM 3 (reads: 1/116), LiM 4 (reads: 0/192), LuM 1 (reads: 0/1268), PT 10 (reads: 0/189), PT 11 (reads: 0/925)
- *WBSCR17* (chr7q11.22__70597468__G>C) in samples: LiM 1 (reads: 2/964), LiM 2 (reads: 0/619), LiM 3 (reads: 1/1321), LiM 4 (reads: 1/374), LiM 5 (reads: 0/300), PT 11 (reads: 2/957)

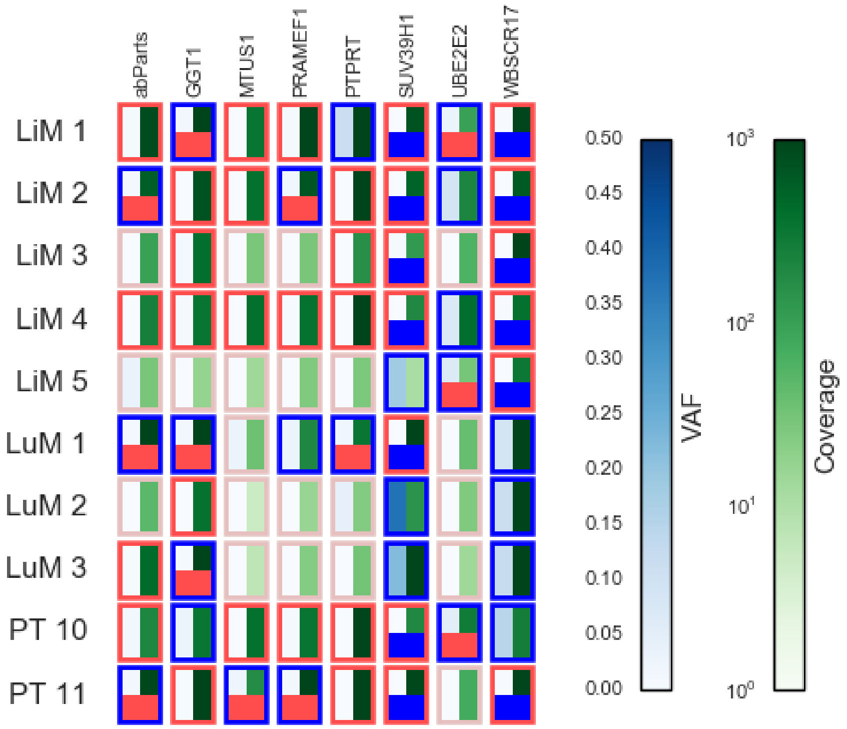


**Mutation patterns of putative artifacts in patient Pam03.** Treeomics identified 26 putative artifacts (out of 880 investigated variants; 3.0%). Additionally there were 55 putative false-negatives due to insufficient coverage (unknowns; data not shown). The color of the border of each rectangle representing a variant illustrates the original classification, the color of the left bar within each rectangle illustrates the VAF, and the color of the right bar illustrates the coverage. If a variant was identified as a putative artifact, a smaller rectangle with the changed classification color is added on top of the bars. Blue borders correspond to variants classified as present, red absent variants, and light red unknown mutation status.

*Treeomics settings:* sequencing error rate e: 0.005, prior absent probability c0: 0.5, max absent VAF: 0.05, LOH frequency: 0.0, false discovery rate: 0.05, false-positive rate: 0.005. Absent classification minimum coverage: 100.

© Treeomics 1.5.2, Aug 31, 2016
