# Supplementary figures and images for: Reconstructing metastatic seeding patterns of human cancers
Source: Nat Commun. 2017 Jan 31;8:14114. doi: 10.1038/ncomms14114 (PMC5290319; doi:10.1038/ncomms14114)

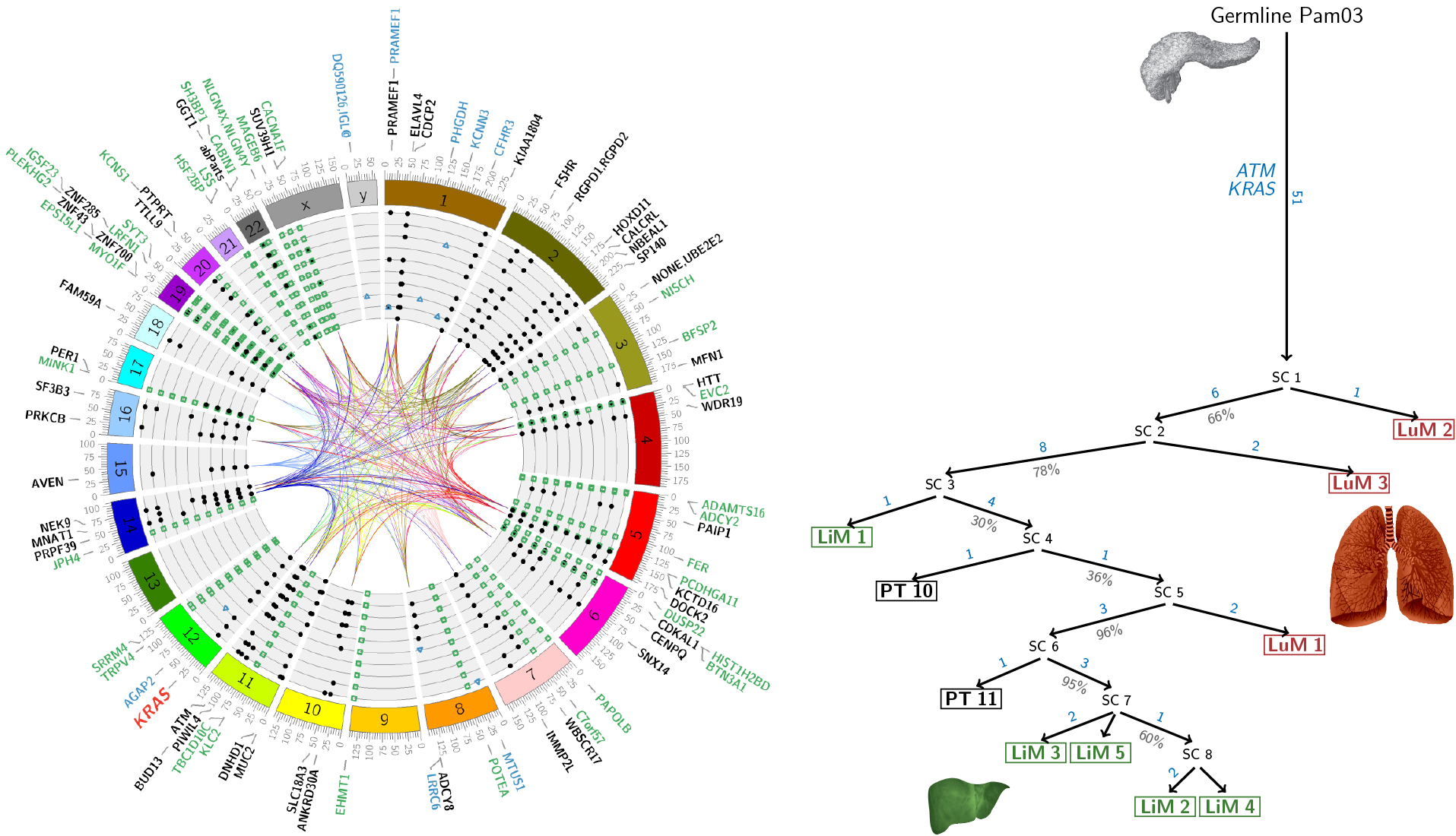

Supplement: Supplementary Software 1 — Treeomics v1.5.2 [file ncomms14114-s5.zip › Treeomics_v1.5.2/repository_illustration.png]
